# Recent advances on anti-scarring properties of amniotic membrane: emerging strategies and clinical potentials in regenerative medicine

**DOI:** 10.1186/s13287-026-05126-x

**Published:** 2026-06-25

**Authors:** Kiarash Soltani, Farnaz Niknejad, Shayan Sadrinasab, Yasaman Gholinezhad, Radman Mazloomnejad, Tahereh Tayebi, Hassan Niknejad

**Affiliations:** Department of Pharmacology, School of Medicine, Shahid Beheshti University of Medical Sciences, Tehran, Iran

**Keywords:** Amniotic membrane, Scar, Angiogenesis, Inflammation, Scarless wound healing, Wound healing

## Abstract

Scarring remains an inevitable consequence of adult wound healing, often accompanied by functional, aesthetic, and psychological burdens. In contrast, fetal wound healing follows a fundamentally different trajectory, characterized by minimal inflammation, abundant hyaluronic acid, elevated type III collagen, and balanced matrix remodeling, culminating in regeneration without scar formation. This regenerative capacity is largely absent in adult tissues, which is the main incentive for understanding the pathophysiology of scarless healing phenotype to replicate such outcomes in adult tissues. The human amniotic membrane (hAM), as an embryogenic derivative, shares many structural and biochemical properties with fetal cutaneous tissue, positioning it as a promising biological tool to bridge adult wound healing toward a scarless, fetal-like outcome. hAM contains a rich milieu of growth factors, anti-inflammatory cytokines, and extracellular matrix components that support epithelialization, angiogenesis regulation, fibroblast modulation, and myofibroblast suppression. These effects are mediated through modulation of key signaling pathways including TGF-β/SMAD, PI3K/AKT, Wnt/β-catenin, and MAPK cascades; each central to the orchestration of fibrosis and regeneration. Furthermore, hAM’s angio-modulatory behavior, antioxidant capacity, and context-dependent immune regulation contribute to a healing microenvironment that more closely mimics fetal conditions. In this review, we highlight the unique biology of fetal scarless healing which can be harnessed through the use of amniotic membrane-based therapies in adults. We highlight how the properties of hAM can help shift adult wound healing toward a more regenerative, less fibrotic outcome. We also examine different ways hAM is processed and applied in clinical settings and processing effects on anti-scar characteristic of hAM and discuss the main challenges that still need to be addressed, such as product standardization and optimizing treatment protocols, in a biomimetic way from scarless wound healing in embryo. Altogether, application of hAM presents a compelling and biologically sound approach to promote scarless wound healing in adult patients, a longstanding goal in regenerative medicine.

## Introduction

Skin, our largest organ, provides a life-sustaining interface as the first line of defense against external insults, thus playing a crucial role in survival and homeostasis of the human body [1]. Being the most vulnerable and exposed organ, the integumentary system is frequently damaged and skin wound healing process in adults is inevitably concluded with the formation of scar tissue [2]. In developed countries approximately 100 million people per year acquire cutaneous scars from surgical interventions alone which indicates high prevalence of scars globally [3]. Scars can be associated with partial or complete loss of function [4], increased economic burden due to wound care costs [5], and decreased quality of life and psychological burden due to aesthetical concerns [6]. Given the indispensable survival roles of the skin, clinical, aesthetic, and economic burdens of scarring, proper induction of scarless wound healing can have remarkable clinical benefits.

In spite of advances in understanding scar formation process, scar treatment still remains difficult to conduct and nearly impossible to prevent. Common therapeutic approaches to scar, incorporate a variety of methods, with compression therapy [7], silicone gel sheets [8], corticosteroid injections [9], and surgical interventions [10], being well-documented with more available evidence supporting their efficacy with other novel methods, such as gene therapy, radio therapy, cryotherapy, and intralesional injections, still being evaluated in preclinical and clinical studies [11–15]. All of these modalities, although effective to some extent, often lead to futile and unfavorable outcomes [16]. Given the multi-factorial nature of scar tissue development, a multimodal therapeutic approach is most likely the best alternative to successfully minimize and prevent normal and pathologic scars from developing [17]. Moreover, due to shortcomings of previous therapeutic modalities, emerging strategies and novel techniques such as regenerative medicine are being investigated for better scar moderation.

Regenerative medicine is an emerging multidisciplinary field focusing on the regeneration of well-differentiated tissues and organ systems in which some clinical limitations are tried to be overwhelmed by merging various techniques [18]. Given the incapability of current therapeutic modalities in scar prevention and treatment, high morbidity of scar tissue, and growing economic and health burdens of scars, innovative concepts of regenerative medicine and tissue engineering may hold a great promise in inducing scarless wound healing [19].

Human amniotic membrane (hAM), as one of the earliest biomaterials with distinctive features, holds a great potential for use in tissue engineering and regenerative medicine with its initial therapeutic application at John Hopkins hospital, dating back to the early 20th century as a skin substitute to accelerate the treatment of open wounds [20]. In addition to its application in dermatology, AM efficacy has been clinically studied in various medical fields, such as, urology, cardiology, nephrology, orthopedics, and ophthalmology resulting in substantial clinical data supporting the benefits of amnion in tissue recovery [21–30].

**Fig. 1:**
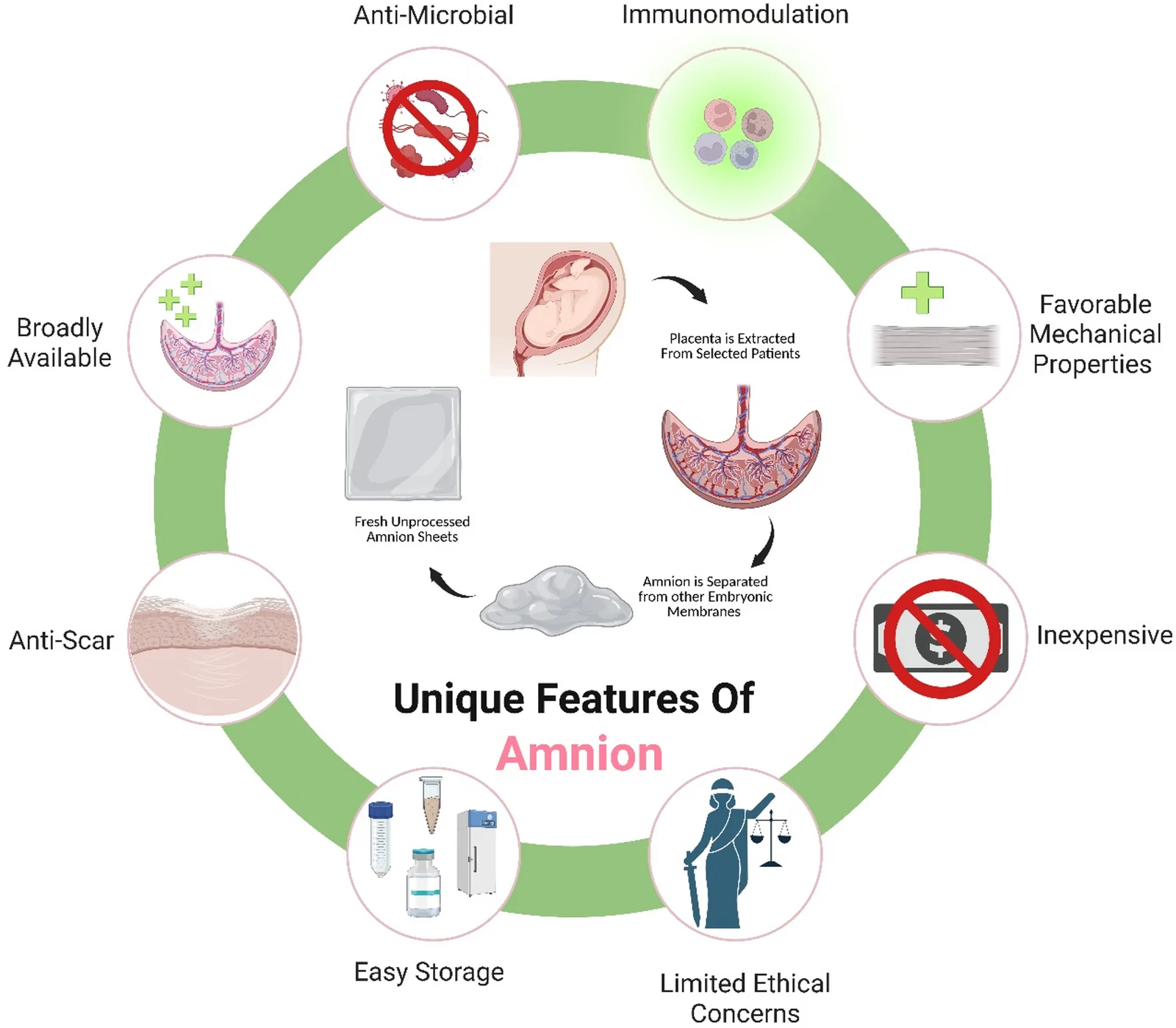
Amnion as a regenerative biomaterial with distinctive features. Created with BioRender.com. Permission for publication was obtained from BioRender.

hAM possesses anti-microbial and immunomodulatory functions (Fig. 1); the features become more prominent when considering that infection and inflammation are profound drawbacks in scar free wound healing [31–33]. In addition, hAM has low immunogenicity and does not provoke the host immune system when properly applied [34]. Apart from its wide range of biological properties, hAM has favorable mechanical properties, is broadly available, inexpensive to process, can be easily stored, and its application lacks limited ethical concerns [35, 36]. Perhaps the most noted feature of hAM, due to high stem cell and growth factor content, is its anti-scarring property which has great clinical applications in various medical fields [35].

In this review we intend to describe the process of skin injury, wound healing and scar formation and provide the latest clinical and experimental data on advances in inducing scarless wound healing by utilizing the amnion as an embryonic derivative and its related products, as a biomimetic platform that recapitulates key molecular, cellular, and immunomodulatory features of fetal scarless wound healing. We also aim to deliver a deeper insight into clinical efficacy, and safety of AM-derived products.

### Processes of skin injury, wound healing, and scar formation

Skin wounds are caused through a variety of reasons namely burns, surgical interventions, diabetic vasculopathies, and wide spectrum of traumatic injuries, ranging from superficial abrasions to deep blunt or penetrating injuries [37]. Wound healing is a dynamic process designed for restoring tissue integrity after injury and is considered as one of the most complex processes in the human body, as the involvement of inflammatory and regenerative factors in this process is done simultaneously and in close proximity [38]. Despite the precise coordination of different factors and cell lines in ensuring the adequacy of healing, this process often leads to scar formation, which may result in cosmetic or functional impairments.

Although the wound healing process is a highly orchestrated sequence of events, it frequently culminates in the formation of scar tissue. This fibrotic outcome is the result of an imbalanced extracellular matrix deposition, prolonged inflammation, and sustained myofibroblast activity. Scar formation is a highly intricate biological process initiated by tissue injury and orchestrated through a dynamic interaction among various cell types, including fibroblasts, keratinocytes, endothelial cells, and immune cells. It involves a complex cascade of molecular signals, including cytokines, growth factors, and extracellular matrix (ECM) remodeling enzymes. Scar formation is considered as the continuation of wound healing and these two physiological processes are closely intertwined [39]. Understanding the dynamics of wound healing is therefore essential to comprehend the mechanisms underlying scar formation. Wound healing is divided into four sequential yet overlapping phases of hemostasis, inflammation, proliferation, and remodeling [40].

Each of these phases is driven by the coordinated activity of multiple cell types. Neutrophils are among the first responders, providing antimicrobial defense and initiating the inflammatory response. Macrophages play a central regulatory role by transitioning from a pro-inflammatory (M1) to a pro-reparative (M2) phenotype, thereby orchestrating tissue repair and resolution of inflammation. Fibroblasts contribute to extracellular matrix deposition and remodeling, while keratinocytes are essential for re-epithelialization. Endothelial cells support angiogenesis, which is critical for nutrient delivery and tissue regeneration. Dysregulation of these cellular interactions can ultimately lead to excessive fibrosis and scar formation [38, 41].

In adult skin, scarring often leads to the deposition of disorganized collagen bundles, loss of adnexal structures, and reduced mechanical strength compared to uninjured tissue. There are different factors involved in scar formation; genetic predisposition has been proposed as a contributing factor for excessive scarring [42]. In a cross-sectional study on 1659 Japanese patients with keloids, females predominated males which suggests the role of gender in scarring [43]. Although the exact mechanism behind this difference is not elucidated, hormonal changes have been suggested as a major cause. Certain anatomical sites such as anterior chest, lower jaw, and scapular region are observed to be more associated with scarring as these areas are frequently subjected to mechanical load and tension [44].

The major scar types include (1) hypertrophic scars, characterized by raised lesions confined to the wound margin and typically associated with prolonged inflammation and elevated TGF-β signaling [45]; (2) keloids, which extend beyond the original injury boundaries due to fibroblast hyperactivity, excessive extracellular matrix deposition, and persistent TGF-β1 activity [46]; (3) atrophic scars, defined by dermal and subcutaneous volume loss resulting in depressed lesions, often due to insufficient fibroblast activation and reduced collagen synthesis [47]; and (4) contracture scars, which form under significant tension and are associated with sustained myofibroblast activity and ECM remodeling, frequently impairing mobility and function [48] (Table 1).

Scarring is orchestrated by several interconnected signaling pathways; the central pathways associated with scar formation are the TGF-β/SMAD pathway, the MAPK/ERK pathway, the PI3K/AKT/mTOR pathway, and the Wnt/β-catenin pathway. Each plays a unique but overlapping role in promoting or regulating fibrosis. These signaling pathways do not act in isolation but rather form a complex, interdependent network. The persistent activation or dysregulation of one or more of these pathways—particularly TGF-β/SMAD in synergy with mechanical stress sensors and growth factor-mediated cascades—results in excessive matrix deposition and pathological scar formation.

Understanding the molecular and cellular mechanisms driving pathological scar formation is essential for developing targeted and effective therapeutic strategies and offers hope for more effective and durable interventions. The last three stages of wound repair (inflammation, proliferation, and remodeling) can be considered as therapeutic targets for clinical usage, due to their manipulative feature. Therefore, we discuss here the influence of amnion and its derivatives on these phases, given that scarring is largely absent during fetal development comparison of fetal and adult wound healing may offer novel insights.

**Table 1:** Comparative summary of scar types highlighting their dominant signaling pathways, cellular dynamics, and extracellular matrix (ECM) characteristics

Scar type	Key signaling pathways	Cell behavior	ECM characteristics
Hypertrophic	↑ TGF-β1, IL-6, PDGF	Persistent Myofibroblasts	Excessive Collagen Bundles aligned Parallel to the Epidermis
Keloid	↑↑ TGF-β1, IGF-1, VEGF	Hyperactive Fibroblasts, Low Apoptosis	Thick, Hyalinized Collagen bundles with Haphazard Pattern
Atrophic	↓ TGF-β1, VEGF	Fibroblast Deficiency	ECM loss, Sunken Appearance
Contracture	↑ TGF-β1, Mechanical Tension	Overactive Myofibroblasts	Collagen + Contractile Proteins

This framework illustrates how variations in molecular signaling and fibroblast behavior contribute to distinct structural and clinical outcomes in wound healing [49–53]

### Insights into differences in fetal and adult wound healing and their implications for scarless wound healing

In 1971, the concept of scarless wound healing was introduced for the first time through the experiments conducted on fetal lambs, where healing before 120 days of gestation occurred without scar tissue formation [54]. This exceptional regenerative ability was also later observed in human fetuses in 1979 [55]. Initially, it was believed that the unique intrauterine environment, characterized by sterile amniotic fluid, minimal inflammation, reduced mechanical stress, and distinct growth factor profiles, was the decisive factor. However, studies with transplantation methodologies revealed that intrinsic properties of the fetal tissue itself play a more significant role [56, 57]. For instance, when adult skin was transplanted into a fetal wound-healing microenvironment, scarring still occurred, whereas fetal skin transplanted into an adult microenvironment retained its scarless phenotype [58–60]. Ultimately, these findings underscore the importance of intrinsic tissue attributes over extrinsic environmental factors in determining healing outcomes. Scarless wound healing, particularly in fetuses, has been linked to reduced inflammation, organized layout of extracellular matrix (ECM) remodeling, and abundance of type III collagen and hyaluronic acid. These properties create a regenerative microenvironment, distinct from the adult wound environment, where mostly prolonged inflammation and excessive type I collagen deposition often leads to scarring [57, 61–63] (Tabel 2).

**Fig. 2:**
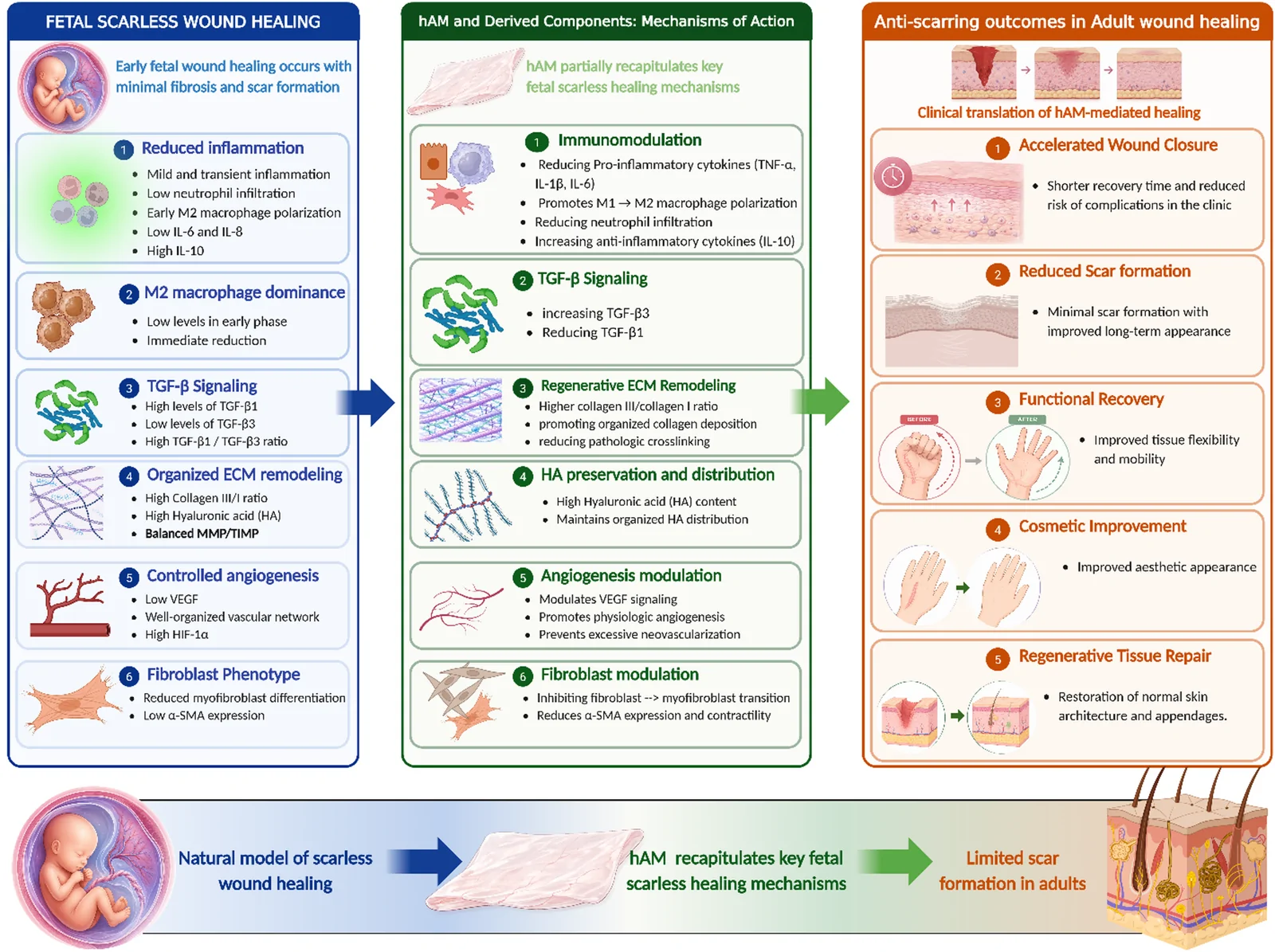
Conceptual framework illustrates how human amniotic membrane (hAM) and its derivatives partially recapitulate key mechanisms of fetal scarless wound healing and shift adult wound repair toward a more regenerative, less fibrotic phenotype. Fetal scarless healing is characterized by reduced inflammation, balanced TGF-β signaling, organized extracellular matrix remodeling, controlled angiogenesis, and regenerative fibroblast activity. Through its cellular components, extracellular matrix, growth factors, and extracellular vesicle-associated signaling molecules, hAM modulates these pathways to promote anti-scarring and regenerative outcomes in adult wound healing. Created with BioRender.com. Permission for publication was obtained from BioRender.

Interestingly, the amniotic membrane, which originates from the fetal environment, exhibits characteristics that mimic these regenerative properties. The membrane is rich in hyaluronic acid, fibronectin, and type III collagen, which are critical for scarless healing. It also contains anti-inflammatory cytokines and growth factors such as TGF-β3, which are known to promote regenerative processes similar to those seen in fetal wounds [19, 64–66]. Studies have highlighted the amniotic membrane’s ability to create a healing environment resembling the fetal state. Its application to adult wounds has shown potential in reducing inflammation, enhancing cellular migration and proliferation, and promoting ECM remodeling and scar modulation. These effects mirror the features of fetal wound healing and suggest that the amniotic membrane can serve as a bridge to replicate scarless healing in postnatal tissues. Furthermore, the anti-scarring potential of the amniotic membrane is supported by its ability to modulate inflammatory cytokines such as IL-6 and IL-8 and regulate the activity of matrix metalloproteinases (MMPs)/ tissue inhibitor of metalloproteinases (TIMPs), which are vital for ECM remodeling [61, 67]. This positions the amniotic membrane as a promising therapeutic tool for transforming adult wound healing into a process that more closely resembles the scarless phenotype of fetal healing (Fig. 2).

**Table 2:** Key differences between adult and fetal wound healing and amniotic membrane characteristics

	Adult wound healing	Fetus wound healing	Amnion effect on wound healing
Inflammation [67]	Severe & More Persistent	Mild & Brief	Decrease Inflammation [68]
Hyaluronic Acid [69]	Low levels – Immediate Reduction	High levels – Long presence	Increase Hyaluronic acid [70]
TGF-β [71]	High levels of TGF-β1 & β2 Low levels of TGF-β3	Low levels of TGF-β1 & β2 High levels of TGF-β3	Decrease TGF-β1/ TGF-β3 ratio [72]
IL-6 & IL-8 [73, 74]	High	Low	Reduce IL-6 & IL-8 [75]
IL-10 [76]	Low	High	Increase IL-10 [68]
Collagen [77]	Lower Collagen III/I ratio Higher Crosslinking	Higher Collagen III/I ratio Lower Crosslinking	Increase Collagen III/I ration [78]
MMP & TIMP [61]	Lower MMP/TIMP ratio	Higher MMP/TIMP ratio	Decrease MMP/TIMP ratio [79] Decrease MMP9 [80] Increase MMP2 & MMP9 [81] Decrease TIMP-2 [82]
Neutrophil Count [63]	High	Low	Decrease Neutrophil Extracellular Traps [83]
VEGF [63]	High	Low	Increase VEGF [83]
HIF-1 [84]	Much lower	High	Induce HIF-1α [85]

### Anti-scar effects of amniotic membrane during wound healing

**Fig. 3:**
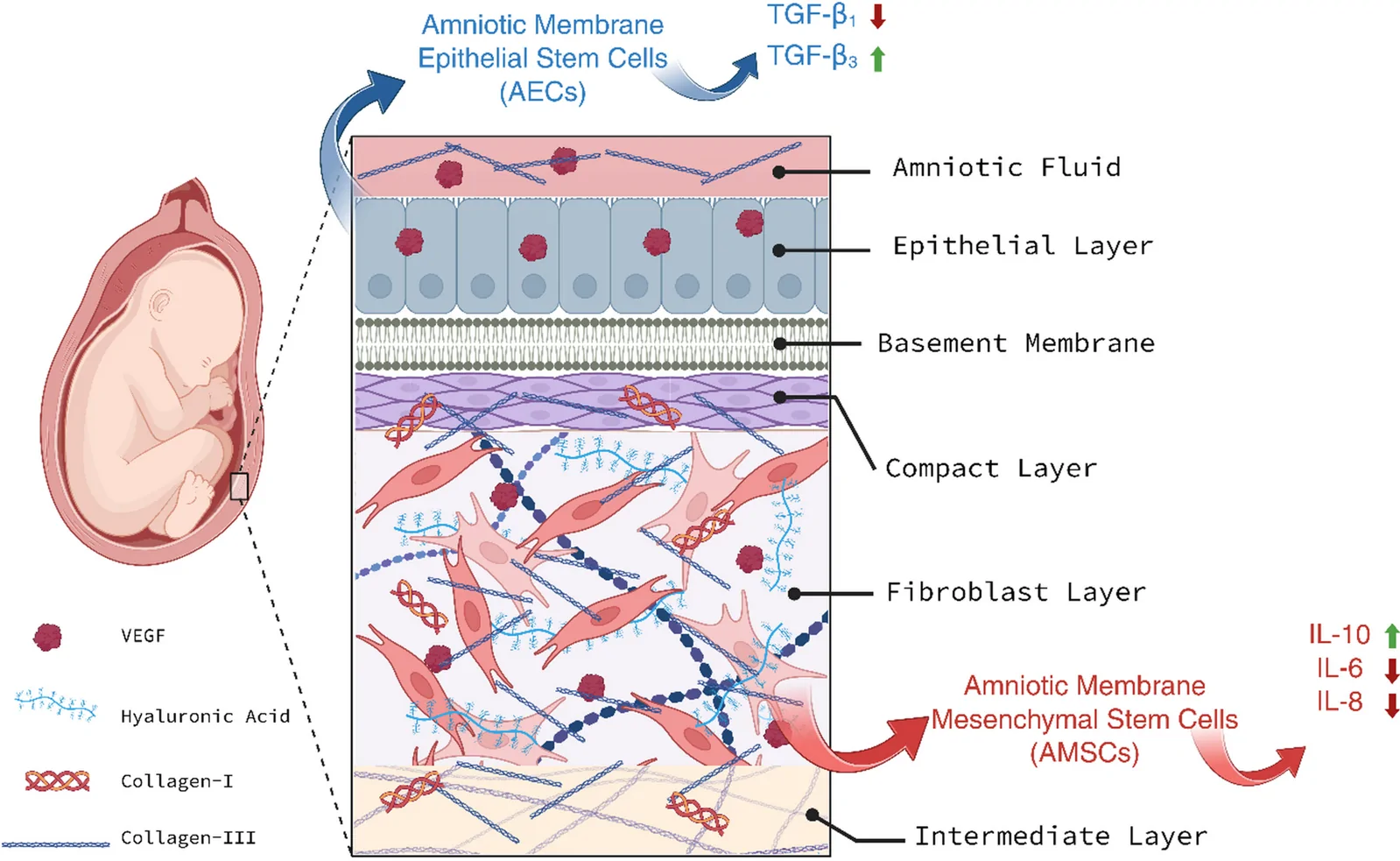
Human amniotic membrane structure and cellular components which affect scarless wound healing. Created with BioRender.com.

The anti-scarring potential of the amniotic membrane can be better understood by examining its multifaceted influence across the sequential phases of wound healing. Acting as a biologically active scaffold, it interacts with inflammatory cells, fibroblasts, and keratinocytes to orchestrate a balanced healing response. Through its rich content of cytokines, growth factors, and extracellular matrix components, the amniotic membrane not only recreates a regenerative microenvironment similar to that of the fetus but also actively modulates processes such as inflammation, angiogenesis, and matrix remodeling (Fig. 2) [86–88]. These coordinated actions can potentially reduce fibrosis and promote tissue regeneration rather than scar formation (Fig. 3). The following sections outline the key mechanisms by which the amniotic membrane exerts these effects during each stage of healing.

In addition to the classical classification of amniotic epithelial and mesenchymal cells, recent single-cell transcriptomic studies have further expanded the cellular landscape of the amnion. These analyses have identified macrophage-associated cell populations within the amniotic membrane, suggesting the presence of immune-related cell subsets alongside the structural compartments. While epithelial and mesenchymal cells remain the primary and functionally dominant components of the amnion, these macrophage-like cells may contribute to its immunomodulatory properties, particularly through regulation of inflammatory responses and macrophage polarization, which are critical for scarless wound healing [89, 90].

**Fig. 4:**
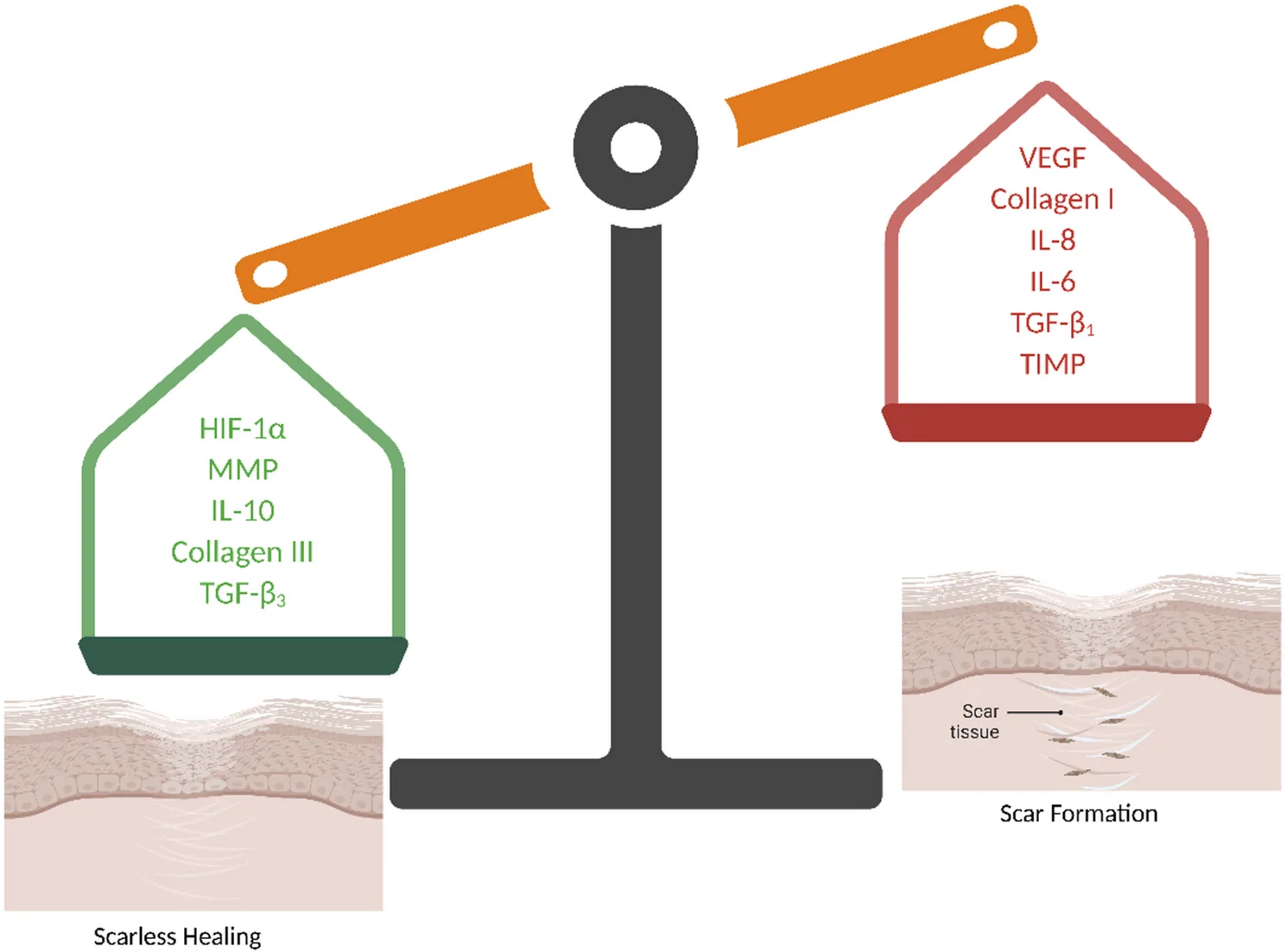
Amnion-derived factors can potentially shift the balance toward a more scarless phenotype. Created with BioRender.com.

I. *Effects of amniotic membrane on wound healing during inflammatory phase*.

Inflammation phase is characterized by the involvement of innate immunity and abundance of neutrophils and macrophages focusing on wound debridement and infection prevention [91, 92]. This acute inflammatory response is essential for clearing debris, pathogens, and damaged tissue, thereby creating a clean environment conducive to subsequent tissue repair [93]. Proper resolution of the inflammatory response is crucial for preventing excessive ECM deposition, and formation of hypertrophic and keloid scars. In several studies on cutaneous wounds with excessive ECM components and hypertrophic scarring, disproportionate numbers of neutrophils as well as neutrophilic retention at the wound site were observed [94, 95].

M1 macrophages, in early stages, express and release TNF-α, IL-6, and IL-1β to induce inflammation and counter infection [96, 97]. Upon the resolution of inflammation in later stages, M1 macrophages switch into an anti-inflammatory phenotype called M2 macrophages [98]. M2 macrophages contribute to neovascularization and blood vessel sprouting by secreting various growth factors such as VEGF [98, 99]. Well-adjusted ratios of M1/M2 macrophages during different phases of wound healing are important for minimizing excessive scarring and other complications of wound healing [100].

The amniotic membrane’s anti-inflammatory and immunomodulatory properties can push the wound through healing phase [101]. Human amniotic mesenchymal cells (hAMCs) and their conditioned medium (CM) have the ability to promote macrophage polarization toward a predominant M2 phenotype. “CM-educated macrophages” were effective in improving tissue regeneration by increasing anti-inflammatory cytokines, such as IL-10 and early-stage form of TGF-β1, while reducing pro-inflammatory cytokines, such as IL-12 and TNF-α [102]. Although TGF-β1 is notably important for wound healing process, uncontrolled levels of this cytokine during later stages of the healing process can lead to chronic inflammation and delayed wound closure with more tissue fibrosis, so its levels must be tightly regulated. The third member of TGF-β family, TGF-β3, has an antagonistic effect on TGF-β1. It attenuates collagen type-l by increasing MMP-9 and reducing fibroblast to myofibroblast differentiation [103]. We recently showed that amnion has potential to lower the TGF-β1/TGF-β3 ratio [72]. In a study by Song et al. it was found that decellularized human amniotic membrane (dHAM) enhances wound healing by reducing the secretion of TGF-β1 on day 3 after scar formation. A lower inflammatory response was confirmed by decreased levels of white blood cells in the dHAM group at various time points [104]. In addition, in an animal study on rats, decellularized human amniotic membrane coated with fibrin (amnion bilayer or AB) showed the ability to reduce the prolonged inflammatory response in full thickness burn wounds. The grafted AB group after seven days of dressing, exhibited lower expression of macrophage inflammatory protein-3α and increased the levels of IL-10, which limit fibrosis, scar formation and help the normal progression of wound healing [105]. Moreover, pathological fibrosis in wound healing was reduced by the use of soluble human amniotic membrane extracts. These extracts downregulate pro-inflammatory cytokines that trigger tissue transglutaminase (TGase2) overexpression, helping to balance immune responses and prevent chronic inflammation. By modulating these pathways, they also reduce the production of rigid ECM structures, contributing to less fibrosis and a lower hypertrophic scarring rate [106]. Tissue transglutaminase is a multifunctional protein inducing ECM stabilization, wound contraction and modulating immune responses during tissue repair; however, its role remains controversial, as TGase2 activity has been associated with both regenerative healing and, in certain contexts, excessive fibrosis and scar formation [107].

Additionally, application of human acellular amniotic membrane (hAAM) combined with exosomes from adipose-derived mesenchymal stem cells (ADSC) demonstrates faster healing and better appearance compared to control group after 7 and 14 days of treatment in mouse diabetic wound model. hAAM-Exos also decreased inflammatory responses and promoted the recruitment of M2 macrophages, resulting in a rapid transition from the inflammatory phase to the proliferation and remodeling phases [108]. In a study by Zhang et al., hyaluronic acid-based hydrogels containing encapsulated amnion-derived conditioned medium (Gel-CM) could modulate immune response. On day seven post injury, the polarization of macrophages toward M2 phenotypes was showed and the expression of proinflammatory cytokines IL-1β and TNF-α was lower in the Gel-CM group [109]. The micronized amnion loaded with self-adhesive hydrogel from the skin secretions of Andrias davidianus (type of salamanders) promoted scarless wound healing through M2 polarization and activation of Wnt pathway. This combination supports hair follicle regeneration, a characteristic of fetal-like healing, and reduced fibrosis [110]. Silver nanoparticle-embedded human amniotic membrane gel showed the same enhancement effect on skin hair follicles in burn wounds of rats by decreasing the inflammatory cells after treatment [111].

Beyond cytokines and cellular modulation, microRNAs (miRNAs) carried by amnion-derived extracellular vesicles (EVs) play a pivotal regulatory role in shaping the inflammatory landscape of wound healing. Sequencing analyses have identified over 300 different miRNAs within EVs from human amniotic mesenchymal stromal cells (EV-hAMSCs), including miR-146a-5p, miR-193b-3p, and miR-100-5p, whose upregulation suppresses inflammatory signaling [112]. Notably, miR-24-3p and miR-146a-5p promote macrophage transition from the pro-inflammatory M1 to the reparative M2 phenotype, while miR-222-3p and miR-181a-5p further support M2 polarization, maintaining a balance conducive to scarless repair. The proportion of M2-related miRNAs was found to be approximately tenfold higher than M1-related counterparts, highlighting the dominant anti-inflammatory property of EV-hAMSC-derived miRNAs [112]. In addition, amniotic membrane proteins (AMPs) may potentially complement this action by modulating key inflammatory pathways by upregulating miR-21 and miR-146 while inhibiting the TLR4/NF-κB pathway, thereby reducing LPS-induced miR-155 and the downstream production of IL-6 and TNF-α (Fig. 4) [113].

Considering that inflammation is a significant drawback in proper healing progression, amnion-based products that modulate inflammation through cellular and molecular mechanisms can give rise to novel therapeutic strategies in the management of chronic wounds and the prevention of scar formation.

**Fig. 5:**
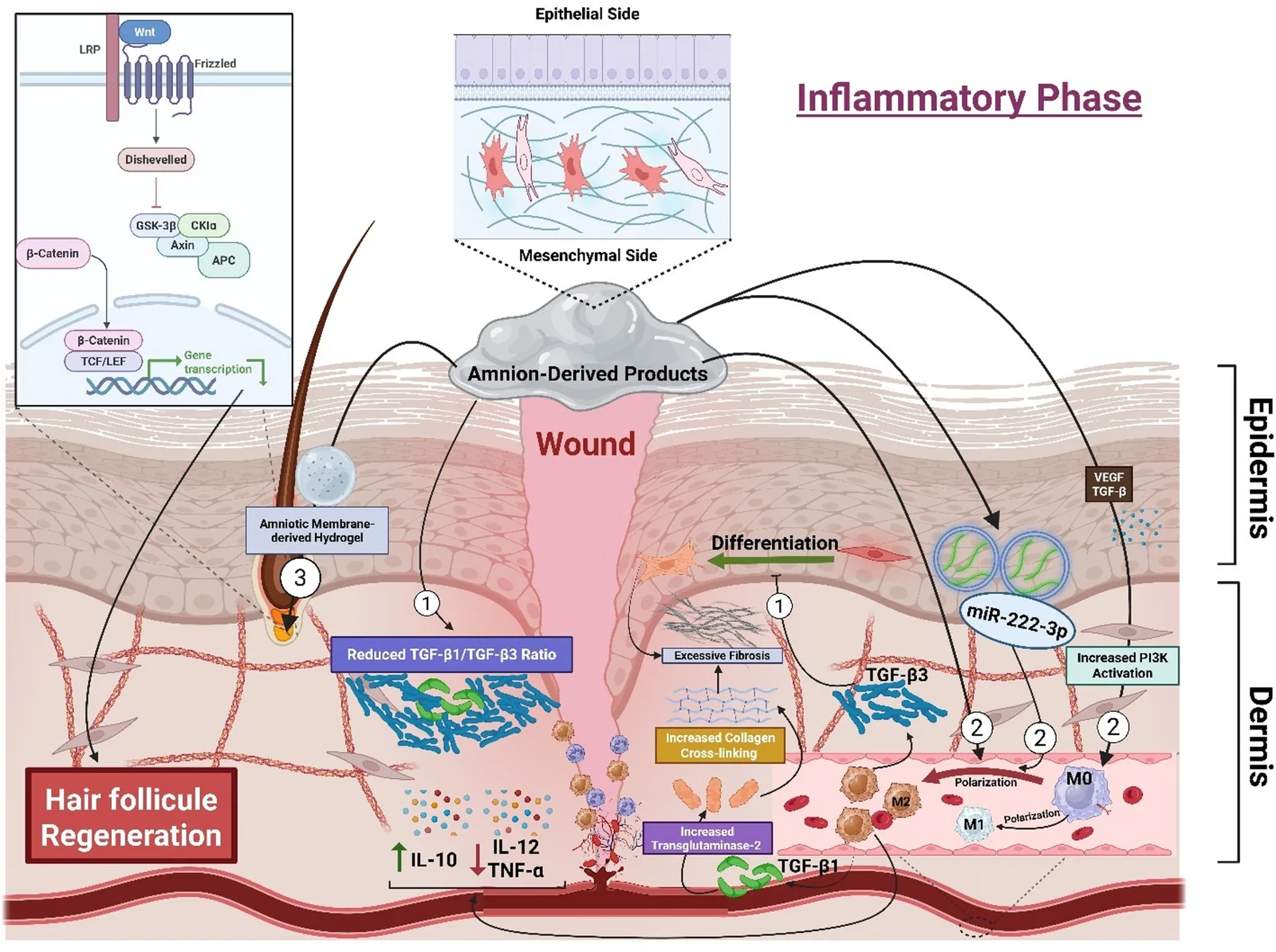
Amnion-derived products play an important role in wound healing by modulating inflammation. 1 They can reduce fibroblast differentiation toward myofibroblast by decreasing TGF-β1/TGF-β3 ratio, thus preventing excessive collagen deposition and aberrant fibrosis. 2 Amnion can also steer macrophage polarization from pro-inflammatory M1 phenotype to anti-inflammatory M2 phenotype. 3 In addition, through the activation of Wnt/β-Catenin pathway amnion can promote hair follicle regeneration following wound healing. Created with BioRender.com. Permission for publication was obtained from BioRender.

II. *Effects of amniotic membrane on granulation tissue and cellular proliferation and angiogenesis*.

The proliferation phase is mainly characterized by the involvement of keratinocytes, fibroblasts, macrophages, and myofibroblasts promoting neovascularization, wound contraction, granulation tissue formation, and re-epithelialization [38]. Recent studies have also indicated that fibroblasts and myofibroblasts which collaboratively deposit collagen in the wound bed, exhibit different phenotypes and heterogeneity with some phenotypes linked to less scaring [97, 114, 115]. Although fibroblast activity and ECM production during this phase is pivotal for restoring structural integrity of the skin, if not properly regulated, can contribute to excessive collagen deposition, imbalanced collagen ratio (I and III), and hypertrophic scars [116, 117].

Different types of amnion ingredients such as amnion-derived cellular cytokine solution (ACCS), dACM, and hAECs, enhance healing in chronic wounds by stimulating epithelialization and promoting the migration and proliferation of keratinocytes and fibroblasts [118–120]. Its beneficial effect on cellular development is further supported by different cytokines and growth factors present within the amniotic membrane, which also possesses fibroblast and keratinocyte-like cell surface markers, such as CD10, CD13, integrin-α1, vimentin and keratinocyte transglutaminase. Moreover, various growth-associated genes in fibroblasts and keratinocytes, including bFGF, IGF-I, IL-6, KGF, GM-CSF, and heparin-binding EGF-like growth factor (HB-EGF), are upregulated by hAEC exosomal microRNAs combination. Besides, the expression of collagen type l and TGF-β1 in fibroblasts decreased following dACM treatment [119, 121]. This may initiate a tendency toward a more regenerative and less fibrotic recovery. While this pattern does not directly confirm a scarless phenotype, it indicates that amnion-derived factors could alter the wound microenvironment in the primary phase of healing, steering it toward a more favorable, fetal-like environment that promotes subsequent stages of skin repair, characterized by balanced fibroblast activity and TGF-β signaling.

The granulation tissue provides a temporary scaffold for the freshly generated tissues until the resolution of the healing process [122]. In an animal study, a hyper dry amniotic membrane (HD-AM) was used as a wound dressing in full thickness third degree skin injury. HD-AM promoted quick and significant granulation tissue development, which was achieved both early and later by prompting and smooth initiation of proliferative phase [123]. Similar results in promotion of granulation tissue growth were also observed with viable (vHAMA) or devitalized (dHAMA) human amnion membrane allografts. vHAMA significantly accelerated re-epithelialization and lowered epithelial gap by day 7, but there wasn’t a significant improvement when dHAMA was applied. However it is noteworthy that dHAMA could still increase granulation formation without viable cells, suggesting that living amniotic cells are not absolutely required for this effect, at least in the early phases of healing in this model [124]. The dHAMA retains elements such as ECM factors, including collagen types I, III, IV, V, and VII, fibronectin, laminin, elastin and hyaluronan, along with a variety of growth factors like EGF, KGF, HGF, and bFGF [125] (Fig. 5).

**Fig. 6:**
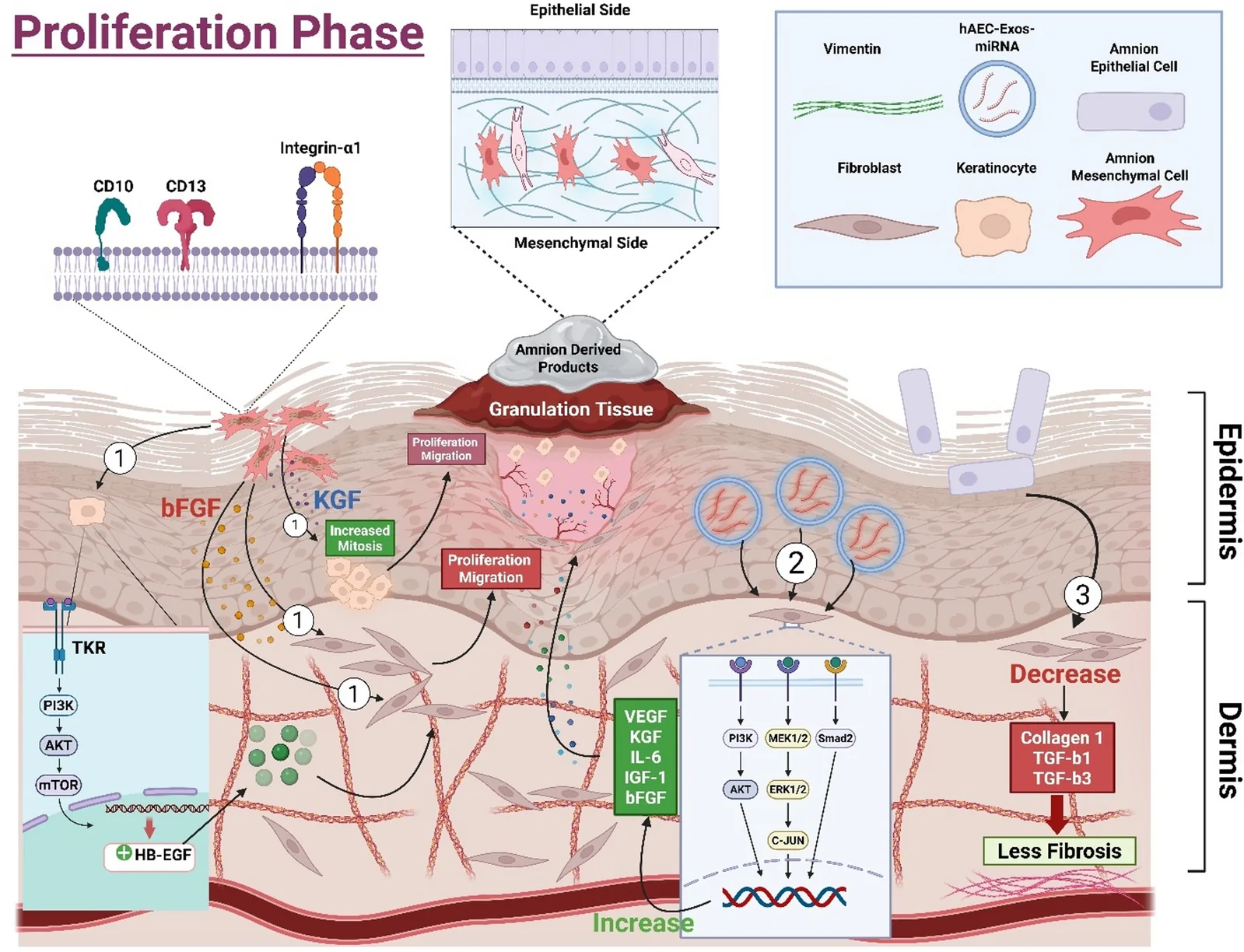
Amnion derived products have the potential to improve the outcome of the proliferation phase. 1 Amnion mesenchymal cells promote proliferation and migration of keratinocytes and fibroblasts by releasing cell proliferation stimulators like KGF, EGF, and bFGF at the early stages of wound healing. 2 Through activation of several pathways, hAEC-Exos-miRNA upregulates growth factor which play an important role in the proliferation phase. 3 Amniotic epithelial cells can also reduce collagen type I, TGF-β1, and TGF-β3 all together leading to reduced fibrosis, and higher proliferation. Created with BioRender.com. Permission for publication was obtained from BioRender.

Angiogenesis is considered a vital process in wound healing as formation of new vasculature is essential for clearing debris and providing oxygen and nutrients for the newly formed and metabolically active tissues. If the process of neovascularization is disrupted during this phase and angiogenic factors remain highly concentrated, vascular maturation is prevented, creating a vicious cycle, that leads to chronic inflammation and healing failure, which is observed in diabetic ulcers. Hypertrophic and keloid scars have been shown to be associated with increased angiogenesis and vascular content [126–128]. Angiogenesis is driven by a plethora of cytokines and molecules, such as placental growth factor (PlGF), vascular endothelial growth factor (VEGF), transforming growth factor β1 (TGF-β1), basic fibroblast growth factor (bFGF), angiogenin, and angiopoietin [129, 130]. Systemic conditions such as diabetes, atherosclerosis, or even aging can interrupt the angiogenic mechanism thus leading to chronic wounds and excessive scar formation [131]. On the other hand, excessive angiogenesis can derail the physiological healing process leading to scar tissue formation. Different studies have established that angiogenesis in scarless wounds is significantly reduced, but with more mature vessels. In conclusion, angiogenesis can be considered as a double-edged sword and a tight balance must be achieved to ensure optimal results [132, 133].

The density and distribution of blood vessels are significantly lower at both keloid and hypertrophic scars compared to normal skin. Paradoxically, although blood flow within scar tissue is elevated, intermittent narrowing of the microvascular lumen, largely driven by endothelial cell hyperproliferation, fibroblast activity, and excessive collagen deposition during the scarring phase, compromises effective perfusion. Consequently, oxygen delivery becomes insufficient, leading to a state of localized hypoxia within the scar tissue [134]. Human-derived placental membranes, especially the amniotic membrane, are rich in regenerative and pro-angiogenic factors such as HGF, IGF-1, VEGF, bFGF, EGF, angiogenin, platelet-derived growth factor B (PDGFB), IL-8 and IL-6 which can greatly influence vascular maturation, motility, and survival [135, 136]. In vitro evaluation of dehydrated human amnion chorion membrane (dHACM) extract on human microvascular endothelial cell (HMVEC) proliferation and production revealed increased endogenous production of more than 30 pro-angiogenic cytokines in HMVEC. Subcutaneous application of dHACM in murine, indicated a dynamic intra-implant neovascular process [131], and its subcutaneous application, increased CD34 + progenitor cells, which are well-known markers of hematopoietic progenitor cells [137]. In an animal model using transplantation methodology, human amnion or chorion mesenchymal stem cells (MSCs) significantly increased blood perfusion and capillary density in hindlimb ischemia model [135].

Importantly, amnion-derived miRNAs also contribute to angiogenic modulation and scarless healing. Certain miRNAs within hAMSC-derived exosomes, such as miR-24-3p, miR-146a-5p, miR-222-3p, and miR-181a-5p, help maintain a regenerative microenvironment by subtly influencing macrophage polarization (previously discussed in inflammation section) [112]. Furthermore, hAMSC-CM and hAEC-EVs regulate VEGF expression in HUVECs via non-coding RNA-mediated pathways, including circ-ABCB10/miR-29b-3p axis, thereby fine-tuning neovascularization to avoid excessive angiogenesis [138]. These vesicular RNAs act in synergy with growth factors to promote organized capillary formation while limiting fibrosis, supporting a fetal-like, scarless healing pattern.

Despite angiogenic properties of amnion, several studies have outlined the anti-angiogenic attributes of amniotic membrane. In vitro evaluation of micronized dehydrated amnion/chorion membrane (µdHACM) extract in tendinopathic injuries was carried out by SE Moreno et al. This study revealed that, depending on the cellular environment, micronized dHACM exhibited different angiogenic properties. Stimulation of angiogenesis was observed in the absence of additional VEGF; however, when VEGF was abundant, as occurs in injured tissue, µdHACM prevented or disrupted excessive vascular network formation. This suggests that µdHACM may help maintain balanced angiogenesis and homeostasis within the wound microenvironment [139]. Additionally, amnion contains multiple anti-angiogenic factors such as IL-10 and IL-1, thrombospondin, TIMP-1, -2, -3 and − 4, endostatin and PEDF [140]. It is worth mentioning that anti-angiogenic feature of the amnion is also intrinsic to this membrane in a side dependent manner which means promotion and inhibition of angiogenesis can be observed simultaneously. In vivo and in vitro, surface dependent induction of angiogenesis by amniotic membrane was previously evaluated by our team. Implantation of the epithelial and mesenchymal sides of the amnion into dorsal skinfold of rodents demonstrated that epithelial side decreased the number of vessel sprouts and vascular length, and by contrast, mesenchymal side increased vessel sprouts and vascularity [141]. This unique property of amnion has also been observed in other studies [136, 142].

It has been shown that both term and pre-term hAECs are capable of inducing angiogenesis in both inflammatory and non-inflammatory environments; however notably under inflammatory conditions, term amnion drives a stronger effect on angiogenic factors such as VEGFA, PDGFB, and FOXC1. Vascular tubules in human umbilical vein endothelial cells (huVECs), which were formed via term hAECs, were longer and more stable in comparison with pre-term hAECs. This research also shows that term hAECs significantly reduced vWF-positive staining, a marker for pulmonary angiogenesis, in injured lungs, indicating mitigated angiogenesis and fibrosis. Therefore, during the early inflammatory phase, amnion effectively delivers pro-angiogenic factors to enhance vascularization at the wound site. However, as healing progresses to the later stages, it helps downregulate these angiogenic components to prevent excessive vessel formation and support scarless healing [136].

As mentioned, a key feature of the amnion is its ability to regulate angiogenesis effectively, whether it is used fresh or cryopreserved. This indicates that the extracellular matrix and structural components of the AM maintain its angiogenic properties. Although certain angiogenic regulators, such as IL-8 and TIMP-2, are reduced in cryopreserved form, its overall angiogenic behavior remains intact [143].

Taken together, these findings emphasize the unique bidirectional role of the amnion in angiogenesis, which varies based on the exposure surface, wound’s stage and even processing method of amniotic membrane. From promoting angiogenesis through its mesenchymal side and secretion of growth factors during the early phases of wound healing, to suppressing immature vascular growth and creating more developed vessels via its epithelial side and reduction of pro-angiogenic components at later stages. This dual functionality makes the amnion suitable for scarless wound healing.

The hypoxic microenvironment during pathological scar formation is an important dynamic phenomenon, closely linked to changes in vascularization. It begins at low hypoxic levels in mild scar stages, progresses to moderate during the proliferative phase, and reaches high hypoxic levels in the regressive stages of scar formation [144]. It is fascinating to know that the amnion shows therapeutic potential under these hypoxic conditions. It accelerates scar healing and enhances fibroblast activity even in low-oxygen environments. Conditioned medium from hypoxically cultured amnion-derived mesenchymal stem cells ameliorates wound closure by accelerating re-epithelialization, angiogenesis and modulating inflammatory components such as IL-1β, IL-6, chemokine ligands 1 & 2, compared to normoxic condition [145]. Although hypoxia typically induces cell apoptosis, various studies demonstrate that amnion cells maintain their traits, including cell surface markers and paracrine growth factors like VEGF and TGF-β, via TGF-β/SMAD2 and PI3K/AKT pathways to regulate cell cycle checkpoints [146]. Decellularized amniotic membrane (dAM) activates the PI3K/AKT pathway, leading to stabilization of HIF-1α and promoting M2 macrophage polarization, which contributes to its anti-inflammatory effects and reduced secretion of pro-inflammatory factors such as VEGF [85, 133]. In keloid tissues, elevated HIF-1α expression may promote angiogenesis and fibroblast activation, contributing to sustained fibrotic activity [147] (Fig. 6).

**Fig. 7:**
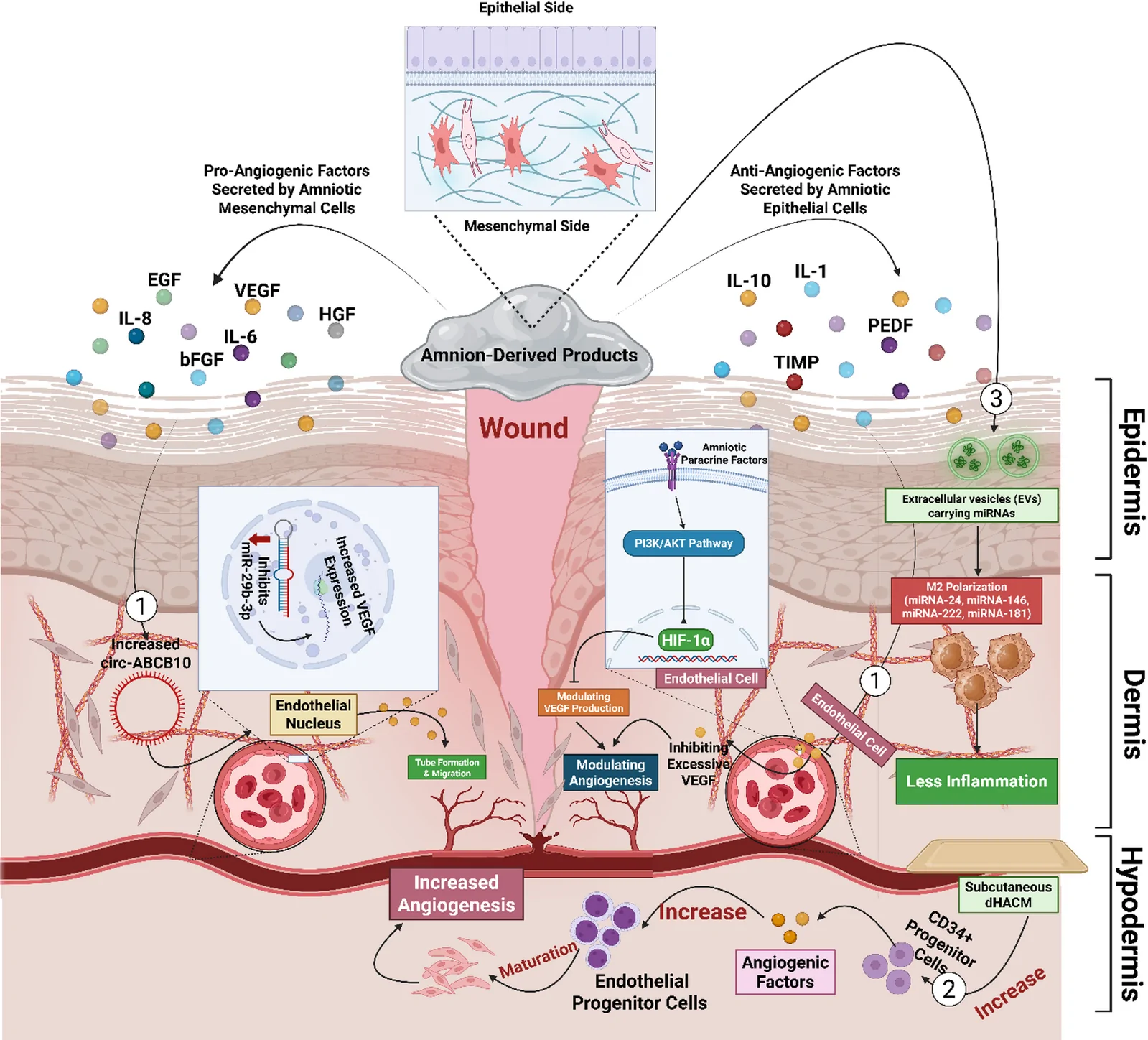
Amnion-derived products regulate angiogenesis during wound healing through coordinated mechanisms [1]. Amnion-derived factors act via the circ-ABCB10/miR-29b-3p axis and PI3K/AKT pathway on endothelial cells, promoting migration, tube formation, and VEGF modulation [2]. Subcutaneous dHACM enhances CD34⁺ progenitor cell recruitment and tissue perfusion [3]. EV-associated miRNAs (e.g., miR-24-3p, miR-146a-5p) promote M2 macrophage polarization, reducing inflammation and supporting scar-limited healing. Created with BioRender.com. Permission for publication was obtained from BioRender.

In summary, amnion-derived miRNAs and other non-coding RNAs integrate with growth factor and hypoxia-mediated signaling to adjust proliferation, granulation, and angiogenesis, capable of supporting a regenerative wound microenvironment conducive to scarless healing.

III. *Effects of amniotic membrane on wound healing during remodeling phase*.

Remodeling phase is crucial for determining the final quality of the scar tissue and can last weeks to years to be fully completed [148, 149]. During this phase the granulation tissue undergoes a series of gradual diminishing changes and myofibroblasts, blood vessels, and immune cells reduce in overall numbers which is the prominent cellular modifications during this phase [148–150]. Noticeably there is a degradation of collagen type III and a gradual replacement of collagen type I through the activity of macrophages and fibroblasts leading to the production of a low cellularity scar tissue [151, 152]. Proper collagen remodeling restores functional integrity of the skin, while disruptions, such as excessive collagen production or insufficient degradation, result in hypertrophic or atrophic scars. Matrix metalloproteinases (MMPs) and tissue inhibitor of metalloproteinases (TIMPs) regulate ECM turnover and remodeling. Imbalance in MMP/TIMP ratio can cause undesirable outcomes. The remodeling phase is thus central to scar formation, as not only it determines the structural and functional integrity of the regenerated tissue but also cosmetic outcomes of the scar is closely related to this phase [152, 153].

Amniotic membrane can modulate fibrosis through the upregulation of tissue regenerative mechanisms such as inhibiting fibroblast differentiation and production of myofibroblast, controlling fibrosis related signals and enzymes, stopping excessive collagen deposition and regulating ECM remodeling process [154–156]. α-SMA is a well-established protein that is used as a biomarker indicating the activation of fibroblast and myofibroblast in various fibrotic tissues and organs [157, 158]. Persistent increased levels of α-SMA, which cause overproduction of ECM and formation of hypertrophic scars, can be hindered through dose dependent freeze‑dried amniotic membrane extract or amnion secreted factors such as conditioned medium from hAECs (AEC-CM), leading to a decrease in differentiation of fibroblasts to myofibroblasts [156, 159]. A novel investigation constructed encapsulated hAECs with alginate-polylysine-alginate (APA), carrying IFN-γ vector within, and examined its effect on hypertrophic scar. The outcome showed it mitigated fibrosis by inhibiting fibroblast transition to myofibroblast, inducing apoptosis in hypertrophic scar (HS) fibroblasts and downregulating ECM components. Furthermore, this product demonstrated better collagen alignment, reduced oxidative stress markers and less inflammation during in vivo assay [160]. In addition, dHACM exhibits anti-fibrotic characteristics by lowering fibrotic substances such as plasminogen activator inhibitor 1 (PAI-1) and connective tissue growth factor (CTGF) in human dermal fibroblasts [161].

Emerging evidence indicates that amnion-derived microRNAs also play a critical role during the remodeling phase by modulating fibroblast activity, ECM deposition, and scar formation. Sequencing of microRNAs in extracellular vesicles of human amniotic mesenchymal stromal cells (EV-hAMSCs) revealed anti-fibrotic and pro-regenerative miRNAs, including miR-150 and miR-194, which target key pathways such as c-MYB and RAC1, leading to decreased collagen deposition and improved fibroblast behavior [36, 112, 162]. hAEC-derived exosomal miRNAs, including miR-23a, miR-27a, miR-203a, miR-34a, and miR-150, regulate myofibroblast differentiation and TGF-β/SMAD signaling, collectively mitigating fibrosis [163–165]. These microRNAs provide a post-transcriptional regulatory layer that complements the protein-mediated effects of amnion, adjusting the remodeling microenvironment to favor scarless healing.

Collagen deposition pattern and collagen types ratio are primarily established during the remodeling phase, both considered central for different scarring phenotypes. Human amniotic membrane shows the capacity to limit the extracellular accumulation of collagen type I, enhance collagen III and reduce overall collagen deposition [106]. In a study by Campelo et al., the impact of the human amniotic membrane (HAM) on the skin healing process in rats were examined. The HAM-treated group showed a well-organized deposition of collagen fibers with a dominance of type III collagen content compared to the control group, indicating a scarless phenotype [78]. In a study carried out on the healing potential of adipose-derived mesenchymal stem cells (AdMSCs) in combination with decellularized (dAM) and stromal human amniotic membrane (sAM) as a wound dressing scaffold were evaluated. The treatment groups compared to untreated groups showed better organized and improved collagen remodeling respectively on day 14 and 21 of experiment [166]. Moreover, amniotic membrane by modulating various enzymes such as BMP1, LOX, and TGase2, all critical for collagen maturation and stability, promoted the formation of a collagen matrix that is more susceptible to remodeling and less prone to excessive scar formation [106].

In addition to shaping collagen architecture, the amniotic membrane modulates enzymatic pathways involved in extracellular matrix turnover, particularly through the activity of MMPs and their inhibitors. Matrix metalloproteinases (MMPs) and tissue inhibitors of metalloproteinases (TIMPs) play a pivotal role in regulating ECM turnover and remodeling [167]. Fetal scarless healing is characterized by a high MMP/TIMP ratio (particularly elevated MMP-1, MMP-3, and MMP-9 relative to TIMP-1 and TIMP-2) which facilitates rapid ECM degradation and regeneration with minimum fibrosis [61]. In contrast, adult chronic wounds typically exhibit a low MMP/TIMP ratio due to excessive TIMP expression and/or reduced MMP activity, promoting pathological ECM accumulation and scar formation [168]. These features are not solely related to the presence of MMPs and TIMPs; but they mostly depend on their balanced interplay with each other during different phases of wound healing. For example, some studies suggest that TGF-β1 may promote ECM accumulation by decreasing the levels of MMP2 and MMP9 [169, 170], while another article shows that TGF-β1 markedly attenuates fibroblast cell proliferation and ECM components by elevation the MMP-2/TIMP-2 mRNA ratio [171]. For more instance, high levels of MMP-9 is led to delayed wounds [172], and even in fetal membrane within scar areas following open fetal surgery for myelomeningocele, MMP-9 and TIMP-1 were measured in higher amounts, while conversely, MMP-1 and TIMP-2 levels were reduced [173], or levels of MMP-2 and TIMP-1in hypertrophic scars were elevated in comparison with normal skin [174]. Thus, we can consider that the critical aspect here is regulation of activity and expression of MMPs/TIMPs to a proper amount, aiming to achieve a dynamic balance in the deposition of extracellular matrix, avoiding both excessive degradation and excessive deposition, not just a simple positive or negative correlation [175]. An optimal equilibrium is needed to ensure adequate tissue remodeling.

Various studies indicate different regulatory impacts of amnion on the expression of MMPs and TIMPs. Although, the MMP to TIMP ratio has been reported relatively lower in micronized dehydrated human amnion/chorion membrane, this modulation may still confer therapeutic benefits in highly inflamed wound environments with excessive proteolytic activity, such as diabetic ulcers, by preventing uncontrolled ECM degradation. Consistent with this regulatory role, several studies observed a reduction in the activity of MMP-2 and MMP-9 following amnion application [80, 120]; while, a rise in MMP-9 level was detected in the treatment group in one study which was carried out on a chronic diabetic murine wound model [81], likely reflecting enhanced remodeling features rather than fibrosis. Furthermore, amnion-derived conditioned medium (AEC-CM) modulates MMP/TIMP dynamics by upregulating MMP-1 and MMP-2 alongside TIMP-1 [176], promoting controlled ECM remodeling while inhibiting excessive collagen deposition. Similarly, in a study utilizing human amniotic membrane/electrospun silk fibroin (AM/ESF) bilayer membranes as wound dressing, a balance between MMPs and their inhibitors was achieved, resulting in decreased collagen synthesis and accumulation, thus minimizing scar formation [177]. While fetal-like high MMP/TIMP ratios define scarless healing during fetal development, amnion-based therapies appear to optimize healing through context-dependent rebalancing, suppressing pathological MMP excess in chronic wounds while preserving sufficient degradative capacity for tissue regeneration (Fig. 7).

**Fig. 8:**
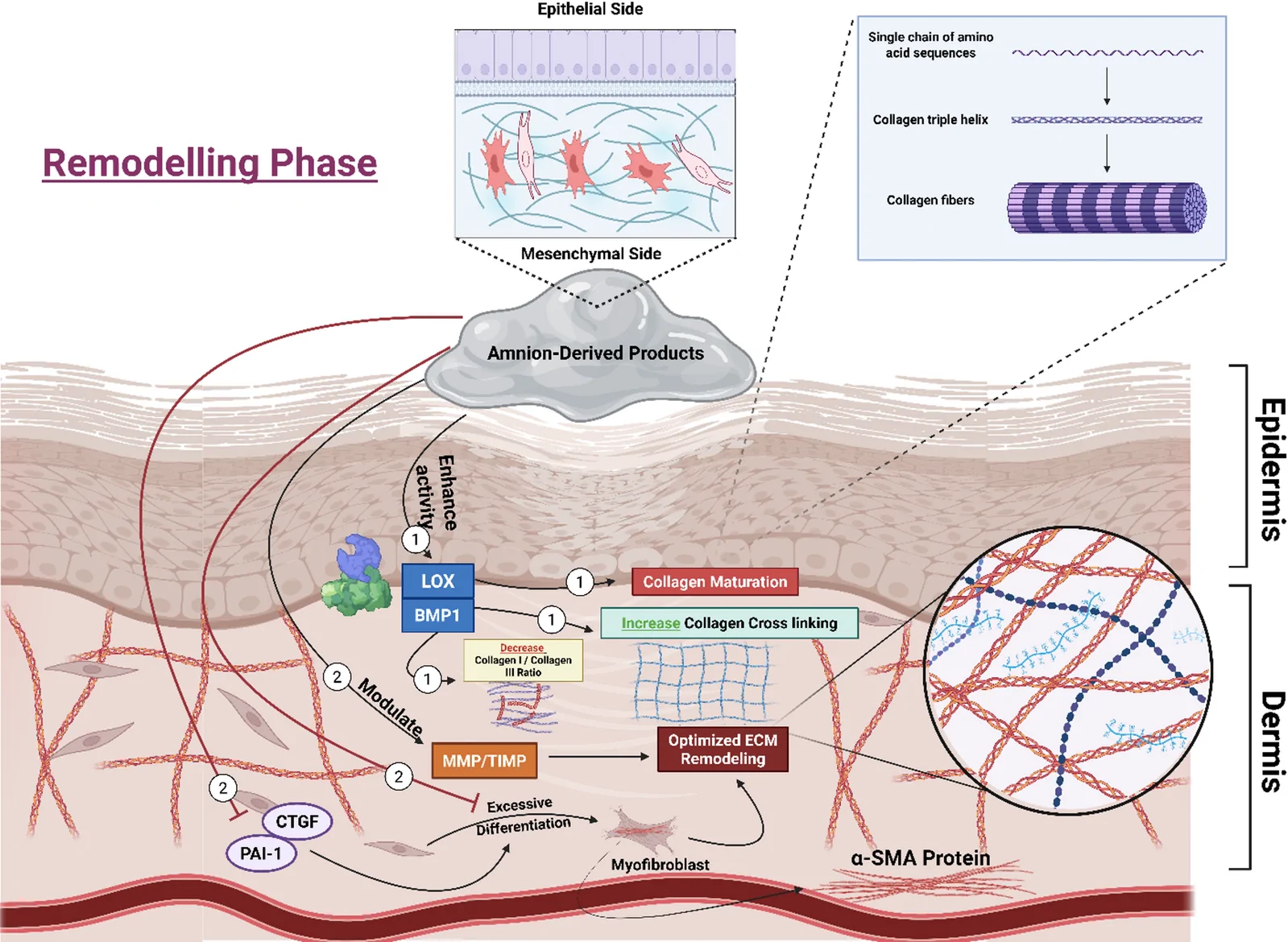
Amnion possess features which can enhance the outcome of the remodeling phase. 1 Amnion can enhance the activity of LOX and BMP1, promoting collagen cross-linking, increasing collagen III/I ratio, and facilitating collagen maturation which all lead to optimized ECM remodeling. 2 In addition, amnion can balance MMP/TIMP ratio, and limit α-SMA production, thus reducing fibrosis and contracture. Created with BioRender.com. Permission for publication was obtained from BioRender.

IV. *Effects of amniotic membrane on key signaling pathways involved in scar formation*.

The amniotic membrane, along with its characteristics that reduce scar formation, operates through various signaling cascades. The TGF-β (transforming growth factor-beta) signaling pathway is the most pivotal in scar development. Upon tissue injury, TGF-β1 is released by platelets, macrophages, and fibroblasts. It binds to its receptors (TGF-βRII and TGF-βRI), initiating the phosphorylation of SMAD2 and SMAD3 proteins [178, 179]. These activated SMADs form complexes with SMAD4 and translocate into the nucleus, where they promote the transcription of pro-fibrotic genes such as collagen types I and III, fibronectin, and α-smooth muscle actin (α-SMA) [180]. In addition, TGF-β also suppresses the activity of MMPs (matrix metalloproteinases), enzymes responsible for ECM degradation, through the upregulation of TIMPs (tissue inhibitors of metalloproteinases) [181]. In normal healing, this pathway is downregulated during the remodeling phase. However, in pathological scars such as hypertrophic or keloid scars, this pathway remains overactive, leading to persistent ECM accumulation and myofibroblast survival [182]. It is demonstrated that human amniotic epithelial cells and their conditioned medium diminish TGF-β/SMAD signaling and lower the differentiation of myofibroblasts and collagen deposition [156, 183]. Similarly, treatment of fibroblasts with dHACM also shows inhibition of TGF-β/ SMAD signaling, resulting in lowered α-SMA levels in myofibroblasts and diminished contractility of scar tissue [161] (Fig. 8).

The TGF-β/Smad signaling pathway is interconnected with Wnt/β-catenin in the promotion of pathologic scar formation [184]. The Wnt/β-catenin pathway also plays a supporting role in fibrosis by modulating fibroblast behavior and extracellular matrix deposition. The Wnt/β-catenin pathway becomes transiently activated after injury when Wnt ligands are released by keratinocytes, fibroblasts, and inflammatory cells bind to Frizzled/LRP receptors. This inhibits the degradation of β-catenin, allowing it to stabilize, accumulate, and then translocate into the nucleus. There, it activates transcription of pro-fibrotic genes causing fibroblast proliferation, wound contraction, and collagen synthesis [185, 186]. It has been demonstrated that micronized dehydrated human amnion/chorion membrane (µdHACM) possesses an inherent capacity to suppress both Wnt and β-catenin signaling pathways and reduce the expression of associated genes such as AXIN2, TCF4 and TCF7 in human dermal fibroblasts [187].

The MAPK/ERK (mitogen-activated protein kinase/extracellular signal-regulated kinase) pathway plays a central role in the proliferation and remodeling phases of wound healing, both critical in scar formation. This pathway is rapidly activated after injury by growth factors such as EGF, FGF, PDGF, and TGF-β1, which bind to receptor tyrosine kinases (RTKs) and induce RAS–RAF–MEK signaling, ultimately leading to ERK activation. Upon activation, ERK promotes fibroblast proliferation, migration, and differentiation into myofibroblasts, key drivers of wound contraction and excessive collagen production seen in hypertrophic scars [188]. Additionally, ERK enhances keratinocyte proliferation, supporting re-epithelialization and epidermal thickening [189]. Through crosstalk with pro-fibrotic signals like TGF-β1, ERK further amplifies fibrotic responses. Excessive ERK activity has been linked to hypertrophic scars and keloids, making MAPK/ERK pathway, potential target of anti-scarring therapies [190]. Co-culture of keratinocytes with human amniotic epithelial stem cells (HAESCs) significantly upregulates ERK, JNK and AKT signaling pathways and promote keratinocytes migration and proliferation proteins such as Cyclin D1, Cyclin D3 and Mdm2 in wound healing process [191]. In addition, human amniotic mesenchymal stem cells (hAMSCs) and their conditioned medium enhance re-epithelialization and protect keratinocytes and fibroblasts from heat-induced apoptosis through coordinated activation of PI3K/AKT and GSK3β/β-catenin signaling pathways, which act downstream in a sequential manner [192].

**Fig. 9:**
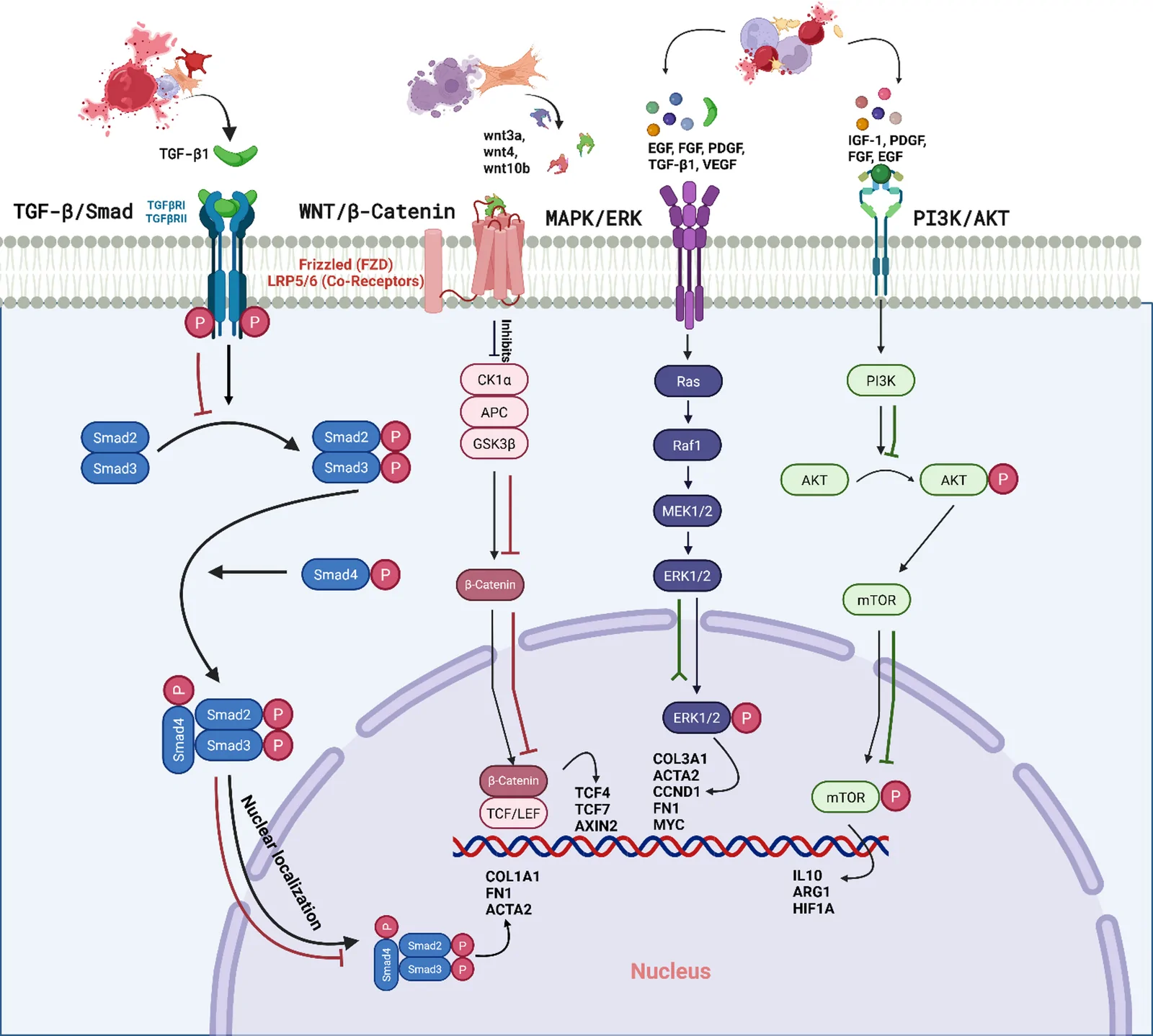
Key signaling pathways in the pathophysiology of scar formation. Red lines indicate the inhibitory effect of amnion-derived products. Green lines indicate promoting roles of amnion-derived products. Amnion-derived products can inhibit TGF/Smad and Wnt/catenin pathway at different levels resulting in reduced scarring. However, these products can also upregulate ERK and PI3K pathways which although it is beneficial to wound healing but can also result in fibrosis. It has been observed that the fibrotic effect of these products is phase-dependent and early administration can be associated with less scarring. Created with BioRender.com. Permission for publication was obtained from BioRender.

The PI3K/AKT/mTOR pathway is another important axis involved in scar formation which is activated by growth factors such as IGF-1, PDGF, and FGF. This pathway plays a major role in fibroblast survival and metabolic activity leading to collagen and ECM deposition. By stimulating angiogenesis and inhibiting apoptotic signals, PI3K/AKT contributes to the persistence of fibrotic tissue, especially in highly cellular scars like keloids and hypertrophic scars [193–195]. Interestingly, pathway activation may have context-dependent effects, as activation during the subacute phase of injury has been shown to reduce scarring and improve functional recovery [196]. The PI3K/AKT pathway also mediates the immunomodulatory effects of the amnion. The release of growth factors such as EGF by dehydrated amnion membrane (dAM) regulate the PI3K/AKT pathway, which then phosphorylates the HIF-1-α pathway, leading to an increase in anti-inflammatory gene expression like IL-10, Arg-1, and the polarization of M2 macrophages, demonstrating the capability of dAM to influence inflammatory conditions during tissue repair [85]. In addition, co-culture of exosomes derived from human amniotic epithelial cells with both human fibroblasts and human umbilical vein endothelial cells (HUVECs) importantly induced migration and proliferation of fibroblasts and a higher capillary density in HUVECs through phosphorylation of PI3K-AKT-mTOR pathway [197]. In addition to modulating intracellular signaling pathways, the amnion exerts protective effects against oxidative stress, further contributing to favorable wound healing and scar attenuation (Fig. 9).

V. *Effects of amniotic membrane on oxidative stress*.

An excessive and unfavorable amount of oxidative stress can result in pathological scarring. For example, it is shown that hypertrophic scars contain a lower amount of ROS scavengers when compared to normal tissue. Therefore, to achieve a scar free wound healing, a balance between ROS production and scavenging must be maintained [198]. Amnion has the ability to act as a scavenger of free radicals and eliminate ROS from its environment [199]. One study that investigated the effect of oxidative stress from H2O2 on dermal fibroblasts alongside human amniotic membrane extract (hAME), revealed protective benefits of (hAME) for re-epithelialization, as well as cell viability and proliferation, while inhibiting cell apoptosis. In addition, hAME reduced oxidative components such as superoxide dismutase and catalase, while promoting anti-oxidant agents like glutathione [200]. Amniotic epithelial cells show greater resistance to ROS stimulation compared to mesenchymal cells by releasing higher levels of antioxidant markers such as Nrf2, HO-1 and SOD2 [201]. While the biological activities of amnion, such as its antioxidative effects, play a central role in wound healing, these properties can also be substantially influenced by the preservation methods applied to the tissue.

### Effect of different preservative methods on anti-scarring features of amnion

Over a century ago simple unprocessed fresh amnion sheets were used as biological dressing skin grafts intended for enhancing cutaneous wound healing [20]. Currently however, various production and processing methods of amnion are available, encompassing membrane isolation, preparation, preservation, sterilization, and amnion cross-linking. Preservation techniques mainly include, Cryopreservation, Lyophilization (Freeze-Drying), glycerol-preservation and, Dehydration (air-drying), each having relative advantages and disadvantages [202–204]. The clinical utility of amnion in scarless wound healing is significantly influenced by how the tissue is preserved prior to application and each technique has distinct impacts on the biological characteristics and regenerative potentials of the amnion. A comparative summary of the biological and anti-scarring effects of different preservation methods is presented in Table 3.

Fresh amnion preserves its trilaminar structure, intact ECM protein profile, high cellular viability, and full spectrum of growth factors, making it the biological benchmark for regenerative wound healing. However, its rapid degradation and limited storage capacity restrict its clinical use [205]. Consequently, preservation strategies inevitably introduce structural and functional alterations, including reduced cellular viability, impaired proliferative capacity, and diminished growth factor content [206].

Cryopreservation technique involves quickly freezing the tissue and has been shown to maintain key structural components of the amniotic membrane, including stromal layers and epithelium, albeit with partial loss of cellular viability (from 97% to approximately 50%). Importantly, cryopreserved amnion retains several critical anti-scarring properties, including anti-inflammatory, anti-fibrotic, and anti-angiogenic effects [206–208]. Experimental evidence suggests that cryopreserved and fresh amnion may exert comparable anti-fibrotic activity, as demonstrated in a liver fibrosis model [209]. Maintaining cellular viability during the cryopreservation process at an acceptable level, is a key strategy that leads to reduced inflammation due to decreased secretion of pro-inflammatory cytokines such as TNF- α and IL-1α and upregulated levels of anti-fibrotic agents like PGE2 and IL-10 [210]. In addition, cryopreserved amnion also emits lower amount of profibrotic cytokines such as angiogenin, TGF-β1, IL-6 and IFN-γ compared to non-preserved amnion [211]. The cryopreservation method as well as fresh amnion, indicates anti-angiogenesis effect in the presence of epithelial layer, but anti-angiogenic factors TIMP-1 and 2, thrombospondin and endostatin are released in lower amounts by cryopreserved amnion in contrast to fresh AM [208, 212]. This controlled regulation of angiogenesis may contribute to a more organized vascular network, resembling fetal wound healing patterns and reducing the risk of excessive fibrosis. Cryopreserved amniotic membrane exhibits higher levels of hyaluronic acid (HA) within the extracellular matrix, with a more uniform and organized distribution, unlike dehydrated human amniotic membrane (dHACM). A similar pattern of HA organization is characteristic of fetal wound healing and is associated with reduced scar formation, suggesting a potential contribution of HA architecture to the anti-scarring properties of cryopreserved amnion [213]. However, cryopreservation requires specialized equipment and storage infrastructure that may not be readily available in all facilities, and its storage duration is generally limited to 12–24 months [35]. Despite these practical limitations, it remains one of the most widely utilized preservation methods due to its ability to maintain key biological and anti-scarring properties.

Lyophilization mostly maintains the histological structure of fresh amnion, but in comparison to cryopreserved amnion, it causes more structural damage and reduction in growth factors, including decrease in EGF, HGF, FGF, and TGF-β1, while expressing reduced immunogenicity due to critical cell loss. However, there is still no significant difference in tissue structure and ECM composition [206, 214]. The comparison between fresh, lyophilized, and cryopreserved amnion products, revealed similar basement membrane protein content in all of the samples through immunohistochemistry of ECM. Although most ECM components are retained after lyophilization, some certain components such as collagen type I, collagen type III (an anti-scarring marker), fibronectin, and laminin are diminished or absent after lyophilization [205, 215]. Nonetheless, lyophilized amnion exhibits more acceptable cell attachment and proliferation for endothelial cells compared with fresh and cryopreserved membranes and also continues to demonstrate an anti-scarring effect, especially at lower dosages [216, 217].

Dehydration-based preservation methods fail to save the amnion’s structure and are associated with greater structural disruption and reduced biological activity since it is a much harsher technique [213, 218]. Dehydrated amnion exhibits compromised ECM integrity and diminished anti-inflammatory effects, and experimental studies have demonstrated limited efficacy in reducing postoperative scarring as indicated in the study by Chun et al., in which dried human AM showed no significant impact on inflammation and subsequent scar formation following strabismus surgery in rabbits [219]. However, despite the damage to amnion integrity, the reduction in immunogenicity resulting from decreased cellular viability, together with the ability for extended room-temperature storage [36], may render dehydrated amnion a practical option in settings where long-term storage and reduced immunogenicity are prioritized.

Glycerol preservation keeps the structure and morphology of the amniotic membrane, at the cost of total loss of cellular viability [206]. A key element of the glycerol preservation method is its ability to decrease water while increasing solute amounts, additionally it maintains the biological properties of the amnion over long time storage, which ultimately leads to a lowered adverse effect from the freezing process [220], while also offering a low-cost approach with antibacterial activity [221]. Glycerol-preserved human amnion demonstrated greater cell attachment stability than both air-dried and freeze-dried forms [222], however it is also noted that it has a dose-dependent growth inhibitory effect. Thus glycerolization requires additional studies regarding the selection of this method as a scar reducing technique [205].

Gamma-irradiation is an additional method alongside other processes such as lyophilization and air-drying to lower microbial burden. It significantly alters the biological performance of the preserved amnion. High-dose irradiation has been shown to disrupt basement membrane integrity and reduce key growth factors, including bFGF, EGF, and TIMP-4, thereby potentially impairing the regenerative capacity. In a study by Paolin et al., it was demonstrated that this cytokine reduction occurred with high doses of radiation, while at lower levels (e.g., ≤ 10 kGy) no significant changes were observed in comparison with fresh amnion [223]. In another study, comparison of irradiated and non-irradiated glycerolized amnion revealed that the irradiated group (25 kGy) demonstrated collagen disorganization and loss of collagen type IV and laminin within the basement membrane, whereas the non-irradiated group retained a thicker collagen layer; a change that may promote scar formation and highlights the importance of dose optimization in utilizing this method [224].

Other processing approaches, such as decellularization, involve the removal of cellular and antigenic components, thereby contributing to immunomodulatory effects with potential to reduce scarring. In vitro studies have shown that decellularized amnion suppresses M1 polarization in LPS-induced macrophages through inhibition of Toll-like receptor (TLR) and TNF signaling pathways, while promoting M2 polarization. Consistently, in vivo studies demonstrate reduced persistent inflammation and decreased fibrous capsule formation following implantation of decellularized amniotic membrane [225]. Although there might be some concerns about the lack of stem cells and growth factors in decellularized amniotic membrane for wound healing process, enriching this structure with other viable cells, like adipose-derived mesenchymal stem cells (AdMSCs), could be seen as an alternative solution [226]. In addition, by keeping the ECM pristine during decellularization, it has the most potential for cell growth in long term in comparison with cryopreserved and lyophilized [205]. Despite some advantages, the biological behavior of decellularized amnion appears to be time dependent. While early inflammatory responses may be attenuated, its prolonged persistence in vivo has been associated with sustained low-grade inflammation, which may ultimately favor fibrotic responses [205]. Overall, decellularization represents a promising immunomodulatory strategy, but its reduced biological activity suggests it may be more effective as a scaffold-based platform rather than a standalone approach for reducing scar formation.

In conclusion, the method of amniotic membrane preservation can change its biological function, including anti-scarring efficacy. While fresh amnion is considered as the gold standard for minimizing scar formation due to its high cellular vitality, full spectrum of growth factors and intact ECM structure, its widespread clinical use is limited by inherent challenges related to storage, handling, availability, along with concerns about immune reactions. Among preservation techniques, cryopreservation offers the most balanced approach by maintaining the structural integrity, anti-inflammatory and anti-fibrotic properties, resembling fresh AM, despite a loss of viability in cells. However, its expensive approach and limitations for prolonged storage, along with immunogenicity issues arising from improved cellular viability preservation, have made it less favorable for clinical application currently. Lyophilization and dehydration offer long term stability and lower immunogenicity; although they cause damage to the ECM organization, the anti-scarring feature is not only related to amniotic cells but also relies on ECM components as well. Nevertheless, achieving an optimal balance between preservation of biological activity and minimization of immunogenicity remains an important consideration. Although current findings are encouraging, further comparative studies are needed to better define the ideal preservation strategy for scarless wound healing applications. Glycerolization also requires additional evaluation, especially since it is typically not employed as an independent technique. It maintains membrane morphology and provides capacity for long-term storage with antibacterial properties; yet, it has not proven to be a good option for scarless wound healing, as it may lead to complete cellular loss and possible growth-inhibitory effects. Decellularization demonstrates promising immunomodulatory properties and may reduce inflammatory and fibrotic responses via macrophage polarization; however, the associated damage to amnion cellular bioactivity may limit its effective capacity for scar reduction on its own. Additionally, adjunct processes such as gamma irradiation also influence outcomes by causing changes in cytokine, ECM structure and immune response.

Taken together, no single preservation technique completely replicates the biological characteristics of fresh amnion for scarless wound regeneration. Each method provides a variety of features, including regenerative properties, immunogenicity, storage capacity, and structural stability. Therefore, choosing the optimal preservation strategy should be influenced by the intended clinical application, storage requirements, and the specific therapeutic goals related to wound healing and scar minimization. Further comparative studies are still required to establish standardized preservation methods that provide the optimal balance between biological efficacy and clinical feasibility for scar reduction.

**Table 3:** Comparison of different amniotic membrane preservation methods and their effects on biological properties and anti-scarring potential

Decellularization	Gamma irradiation	Glycerolization	Air-drying/dehydration	Lyophilization (freeze-drying)	Cryopreservation	Fresh amnion	Preservation Method
Acellular ECM scaffold preserved; reduced collagen I, collagen III, and fibronectin [225]	Basement membrane and ECM disruption; collagen IV/laminin loss [223, 224]	General structure preserved; cellular shrinkage present [227, 228]	Significant ECM disruption; HA and laminin loss [213, 218]	Moderate ECM preservation; reduced collagen III and laminin [206, 215, 216]	Good structural and ECM preservation; higher HA content [213, 216]	Intact trilaminar structure; native ECM preserved [205]	Structural / ECM Integrity
Acellular	harmful to cellular viability, especially at high doses [223]	Non-viable cells [211]	Largely devitalized [229]	Almost non-viable [230]	Reduced (~ 50%) [212]	Highest viability (~ 97–100%)	Cell Viability
Reduced antigenicity and inflammatory cytokines [225]	Reduced bFGF, EGF and TIMP-4 at high doses [223]	Alters growth factor and TGF-β signaling profile [231]	Reduced cytokine retention [213]	Reduced EGF, HGF, FGF and TGF-β1 [206]	Retains key cytokines and anti-fibrotic mediators [212]	Native growth factors retained [205]	Growth Factors & Cytokines
Promotes M2 polarization and reduced inflammation [225]	Reduced biological activity [223]	Reduced biological activity compared with fresh/cryopreserved AM [230]	Limited anti-inflammatory and anti-fibrotic effects [219]	Lower biological activity than cryopreserved AM [206]	Preserves anti-inflammatory, anti-fibrotic, anti-angiogenic and low-immunogenic properties [206]	Strong anti-inflammatory, anti-angiogenic and anti-fibrotic activity [232]	Immunomodulation / Anti-scarring Features
Low immunogenicity; strong scaffold properties	Improves sterility	Low cost; antibacterial effect; prolonged storage [220, 221]	Long-term room-temperature storage [205]	Long-term room-temperature storage [205]	Best overall structural and biological preservation	Closest to native tissue properties	Advantages
Loss of viable cells and reduced biological activity	Major structural and cytokine damage [223]	Growth inhibitory effects and cellular damage [205, 227]	structural disruption and reduced biological activity [218]	Structural damage and bioactive molecule loss	Partial cellular and growth factor loss [212]	Poor long-term storage stability [205]	Limitations

### Clinical use and challenges in anti-scaring property of amnion

The first clinical application of AM was reported over a century ago and since then preparation methods, target tissues, and forms of application have evolved considerably [233]. In the context of dermatologic applications, the amniotic membrane (AM) has demonstrated considerable promise in promoting scarless wound healing due to its unique combination of anti-inflammatory, anti-fibrotic, antimicrobial, and pro-regenerative properties [35]. Rich in growth factors such as epidermal growth factor (EGF), transforming growth factor-beta (TGF-β), and basic fibroblast growth factor (bFGF), AM supports keratinocyte migration, angiogenesis, and re-epithelialization while modulating fibroblast activity to reduce fibrotic remodeling [87]. These mechanisms are particularly beneficial in clinical scenarios where minimizing scar formation is paramount, such as in facial burns, post-surgical wounds, and chronic non-healing ulcers. Several clinical and preclinical studies have shown that AM application can accelerate wound closure, reduce inflammation, and result in more functionally and cosmetically favorable healing outcomes compared to conventional dressings (Table 4). Similarly, a multicenter retrospective study evaluating dehydrated human amniotic membrane (DHAM) in treatment-resistant diabetic and venous leg ulcers demonstrated significant wound volume reduction after repeated DHAM application, further supporting the clinical regenerative potential of amnion-derived products in chronic wound management [234]. Notably, not all clinical studies evaluating amnion-derived products report scar-specific outcomes; several focus instead on wound closure or functional recovery parameters, reflecting ongoing heterogeneity in outcome reporting across clinical trials. A recent preclinical study demonstrated complete and scarless skin regeneration using a bioactive hydrogel derived from *Andrias davidianus* skin secretion (SSAD) loaded with micronized amnion (MA) [110]. In a full-thickness skin defect model, the combination significantly enhanced keratinocyte and amniotic stem cell proliferation, inhibited apoptosis, and promoted the regeneration of cutaneous appendages including vasculature and hair follicles, outcomes rarely achieved in typical wound healing models. Transcriptomic analysis suggested that the observed regenerative effect was mediated by the hydrogel’s rich growth factor content and synergistic interaction with MA, highlighting its potential for future clinical translation.

Despite these advantages, several challenges impede the widespread clinical adoption of AM-based therapies in dermatology. A major limitation lies in the variability of commercially available AM products, including differences in preservation methods (cryopreserved, lyophilized, or dehydrated) and handling protocols, which can significantly influence biological efficacy and structural integrity. Additionally, clinical protocols, such as optimal membrane thickness, frequency of application, and wound-specific usage, remain poorly standardized across studies, complicating the translation of promising results into routine standardized clinical practice. Moreover, while early-phase trials and observational studies are encouraging, there remains a paucity of large-scale, randomized controlled trials providing high-level evidence for AM’s superiority over existing standard-of-care treatments, particularly when scarless wound healing is concerned. Interpretation of the current clinical evidence remains challenging due to considerable heterogeneity among studies. Major limitations include the lack of direct head-to-head comparisons between different amnion-derived products, inconsistent reporting of membrane orientation, dosage, and application frequency, as well as variability in preservation methods and study design. Furthermore, many studies primarily evaluate short-term wound closure rather than long-term scar quality outcomes using validated scar assessment tools such as the Vancouver Scar Scale (VSS) or Patient and Observer Scar Assessment Scale (POSAS). These limitations complicate direct comparison between studies and hinder the establishment of standardized evidence-based clinical protocols for scarless wound healing. Addressing these gaps through well-designed clinical trials and improved product standardization will be crucial for integrating AM into evidence-based dermatologic wound care and scarless skin regeneration.

**Table 4:** Clinical trials evaluating amnion-derived products in skin-related conditions. Outcomes include both scar-specific and non–scar-related endpoints

Study Title	Conditions	Intervention	Reported Outcome	Limitation	ID
Dehydrated Human Amnion/Chorion Membrane (dHACM) for Recovery After Fractionated Ablative CO2 Laser Resurfacing of the Face	Scarring	dHACM	Outcome not yet reported; endpoint includes time to complete epithelialization	Results not yet reported	NCT01995604
A Prospective Randomized Clinical Trial Using Dehydrated Human Amnion/Chorion Membrane (dHACM) in Total Knee Replacement Patients to Reduce Post-operative Scarring.	Scarring	dHACM	Improved range of motion; no direct scar-specific outcome reported	No scar-specific endpoint	NCT02088567
Multicenter, prospective, randomized, controlled feasibility trial to determine the safety and effectiveness of Dehydrated Human Amnion Chorion Membrane (dHACM) plus Control alone for the treatment of second degree burns as assessed by time to healing and scarring	Healing & Scarring	dHACM	≥ 95% epithelialization; scarring evaluated using Vancouver Scar Scale (VSS)	Limited long-term follow-up	NCT02765737
The Comparison of Microneedling Therapy with or Without Amnion Bilayer Sheeting on Post-Burn Hypertrophic Scar Tissue	Hypertrophic Scar Tissue	Microneedling + Amnion	Changes in pain, erythema, skin thickness, and histological features	Small sample size	NCT04995302
A Randomized, Single Blind, Controlled Trial of Clinical Outcomes of Dehydrated Human Amnion/Chorion Membrane (dHACM) in Lumbar Decompression and Microdiscectomy Surgery	Scarring	dHACM	Improvement in Oswestry Disability Index (functional outcome); no direct scar assessment	No direct scar assessment	NCT02300909
Phase 1 Study of Human Amnion Membrane Powder for Enhanced Wound Healing	Wound Healing	Amnion Powder	Incidence of wound closure; no scar-specific endpoint reported	Focused on wound closure	NCT03754218
Pilot Study to Evaluate Clinical Outcomes with the Use of Biovance, a Dehydrated Decellularized Human Amnion Membrane Allograft, Following Keloid Revision Surgery	Keloid Scar	Decellularized Amnion	Recurrence rate of keloid following revision surgery	Pilot study	NCT02521402
Phase I/II Randomized Blinded Safety, Dose-determining Efficacy Trial Comparing Amnion-derived Cellular Cytokine Solution in 3 Different Regimens with Standard Care 0.9% Sodium Chloride in the Topical Treatment of Partial-thickness Burns	Burn wounds	Cytokine Solution (ST266)	Percentage of wound epithelialization over 21 days	No scar-specific outcome	NCT00886470
A Multicenter Prospective Randomized Clinical Trial Using Dehydrated Human Amnion/Chorion Membrane dHACM in Decompressive Craniectomy Patients to Reduce Postoperative Scarring	Scarring	dHACM	Scar formation assessed via Ease of Dissection score and histological evaluation	No product comparison	NCT02033824
A Comparison of the Effects of Bovine Freeze-dried Amniotic Membrane and Hydrocolloid on Epithelialization After Laser Treatments	Epithelization	Amniotic Membrane	Time to epithelialization	Scar quality not evaluated	NCT01895374
The Effect of Amniotic Membrane Application on Post-Cesarean Wound Healing and Cosmetic Outcomes: a Double-Blind, Randomized Controlled Trial	Scar/Keloid Formation	Amniotic Membrane	Incidence of wound complications and scar/keloid formation	Ongoing trial	NCT06770946

## Conclusion and future perspectives

Scar formation remains one of the most persistent challenges in regenerative medicine, reflecting a fundamental limitation of postnatal wound healing. In this review, we highlighted current knowledge on the biological and molecular underpinnings of scarring and the unique therapeutic potential of the human amniotic membrane (hAM) as a biologically active material capable of modulating this process. By comparing adult and fetal wound repair responses, and by integrating data from in vitro, in vivo, and clinical studies, we outlined how the intrinsic anti-inflammatory, anti-fibrotic, and immunomodulatory properties of hAM can recapitulate core elements of fetal scarless healing within adult tissues. Together, these findings position the amniotic membrane as a promising platform for next-generation strategies aimed at preventing or attenuating pathologic scarring.

A key insight emerging from the literature is that hAM exerts its therapeutic effects through coordinated regulation of all major stages of wound healing. By influencing principal signaling cascades implicated in fibrosis, including TGF-β/SMAD, PI3K/AKT, Wnt/β-catenin, and MAPK pathways, hAM can dampen excessive inflammation, promote balanced angiogenesis, enhance cellular proliferation and migration, and guide extracellular matrix deposition toward a more organized architecture. These multifaceted actions underscore the membrane’s versatility as a biological material capable of addressing the complex, multi-step nature of scar formation.

Despite these encouraging findings, several challenges remain before amnion-based therapies can be fully integrated into standardized anti-scarring clinical practice. Current evidence is characterized by substantial heterogeneity in preservation methods, product composition, wound models, treatment protocols, and outcome measures. In addition, many studies focus primarily on wound closure and epithelialization rather than long-term scar quality assessment using validated tools such as the Vancouver Scar Scale (VSS) and Patient and Observer Scar Assessment Scale (POSAS). Variability in membrane orientation, dosage, application frequency, and follow-up duration further complicates direct comparison among studies and limits the establishment of evidence-based clinical guidelines. Addressing these methodological limitations through standardized study designs and well-controlled comparative trials will be essential for advancing the field.

Looking ahead, the most transformative opportunities for achieving true scarless healing may arise from integrating hAM-based therapies with emerging molecular and biomaterial technologies. Among these, three areas including miRNAs, exosomes derived from the amniotic membrane, and hydrogels hold particular promise.

miRNAs from amniotic epithelial and mesenchymal cells act as potent post-transcriptional regulators of fibrosis-related genes such as TGF-β1, COL1A1, and α-SMA. Their ability to attenuate myofibroblast activation and mimic fetal regenerative signatures makes them attractive candidates for targeted anti-scarring interventions. Future work will likely involve engineering miRNA cocktails or delivery vectors tailored to patient-specific fibrotic profiles [235–237].

Exosomes derived from the amniotic membrane serve as natural nanocarriers of bioactive molecules, including miRNAs, proteins, and cytokines. These vesicles can modulate the wound environment by promoting M2 macrophage polarization, reducing inflammation, and supporting re-epithelialization and vascular remodeling. Their ability to engage with key signaling networks such as TGF-β/SMAD and PI3K/AKT makes them ideal candidates for cell-free, off-the-shelf therapeutics. In the coming years, improvements in exosome isolation, scalability, and targeting will be essential for translating this promising tool into clinical-grade products for regenerative medicine [197, 238–240].

Moreover, hydrogel-based amnion delivery systems, particularly those loaded with exosomes and miRNAs, provide a highly adaptable platform for localized and sustained therapeutic delivery. These biomaterials can be engineered for optimal mechanical strength, biodegradability, and responsiveness to the wound microenvironment. They also offer the ability to mimic the three-dimensional architecture of native skin, support cellular infiltration, and enhance the biological activity of their cargo. In future applications, customizable hydrogel systems may be developed to release specific molecular signals in a controlled manner, further enhancing the efficacy of amnion-based therapies in scar prevention [241–243].

Beyond amnion-derived molecular and biomaterial strategies, other emerging regenerative approaches may further contribute to advances in scarless wound healing. Decellularized extracellular matrix scaffolds, nanotechnology-based delivery systems, bioengineering approaches, and three-dimensional bioprinting have all demonstrated encouraging potential in modulating inflammation, enhancing tissue regeneration, and improving extracellular matrix organization. These technologies may also be particularly powerful when combined with biologically active materials such as the amniotic membrane, offering additional avenues for precise control of the wound microenvironment and functional tissue reconstruction [244–247].

In summary combined with advances in biomaterials and regenerative engineering, the human amniotic membrane stands at the intersection of developmental biology, translational science, and advanced biomaterial engineering. Its ability to orchestrate a pro-regenerative wound environment and limit fibrosis offers a compelling path toward redefining current standards of wound care. Continued refinement of amnion-derived molecules, exosome-based therapeutics, and engineered delivery systems will likely accelerate the transition from supportive wound management to targeted, mechanistically informed interventions capable of achieving true scarless healing.

## Data Availability

No datasets were generated or analysed during the current study.
